# Hierarchical Targeting of TREM2^+^ Myeloid Cells via Acid‐Triggered OMVs Reprogram Immunosuppression and Suppress Osteolysis in Bone‐Metastatic TNBC

**DOI:** 10.1002/advs.202517369

**Published:** 2026-04-07

**Authors:** Fanglu Chen, Yucheng Xue, Xiao Ma, Liang Chen, Zhenxuan Shao, Hangxiang Sun, Haochen Mou, Fangqian Wang, Zilong Li, Yechao Shen, Zhaoming Ye, Xiaohua Yu, Shengdong Wang

**Affiliations:** ^1^ Musculoskeletal Tumor Center Department of Orthopedics The Second Affiliated Hospital of Zhejiang University School of Medicine Hangzhou P. R. China; ^2^ Institute of Orthopedic Research Zhejiang University Hangzhou P. R. China; ^3^ Key Laboratory of Motor System Disease Research and Precision Therapy of Zhejiang Province Hangzhou Zhejiang China

**Keywords:** immunotherapy, myeloid‐driven immunosuppression, osteoclast, TREM2, triple‐negative breast cancer bone metastasis

## Abstract

Triple‐negative breast cancer (TNBC) bone metastasis is characterized by an immunosuppressive microenvironment dominated by triggering receptor expressed on myeloid cells 2 (TREM2) ^+^ myeloid cells, which promote osteoclastogenesis, as well as T cell exclusion. To disrupt myeloid‐driven immunosuppression, we developed a hierarchical targeting nanoplatform (siTREM2@ETP‐PEOz‐OMVs) that exploits pH‐responsive outer membrane vesicle (OMV) exposure for selective myeloid cell uptake in acidic TNBC bone metastatic microenvironment, enabling TREM2 silencing‐driven macrophage repolarization and osteoclast inhibition to alleviate immunosuppression and block tumor progression. After achieving specific targeting of metastatic lesions through phage display‐identified TNBC‐targeting peptides, this nanoplatform utilizes the acidic tumor microenvironment (TME) to trigger pH‐responsive dissociation of 2‐ethyl‐2‐oxazoline (PEOz). The released OMVs subsequently leverage their inherent Toll‐like receptor 4 (TLR4) affinity to achieve selective internalization by myeloid cells. TREM2 silencing repolarizes over 50% of macrophages to a proinflammatory phenotype, activates antitumor immunity via approximately twofold increased CD4^+^/CD8^+^ T cell infiltration, and inhibits over 70% of osteoclastogenesis, thereby achieving 79.5% suppression of osteolysis‐mediated tumor progression. This strategy represents a novel approach for precise myeloid reprogramming in TNBC bone metastasis.

## Graphical abstract

This study developed a hierarchical targeting nanoplatform, siTREM2@ETP‐PEOz‐OMVs, against triple‐negative breast cancer (TNBC) bone metastasis. It precisely delivers therapeutic siTREM2 to monocytes/macrophages within the metastatic niche. This intervention dually regulates cell fate: reprogramming immunosuppressive macrophages and inhibiting osteoclast differentiation. Consequently, it successfully remodels the antitumor immune microenvironment and disrupts the critical osteolytic‐immunosuppressive crosstalk, offering a promising strategy for treatment.

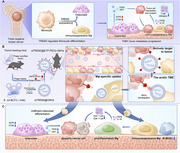

## 1 Introduction

Triple‐negative breast cancer (TNBC) is a highly aggressive and molecularly heterogeneous subtype with poor prognosis, and bone metastasis represents a major driver of patient mortality [1, 2, 3]. The bone microenvironment provides a favorable niche for tumor metastasis, where myeloid cells, such as tumor‐associated macrophages (TAMs) and myeloid‐derived suppressor cells (MDSCs) dominantly infiltrate the bone metastatic niche, establishing immunosuppression via TAM‐secreted interleukin 10 (IL‐10)/TGF‐β that dampen T cell function, as well as MDSC‐driven arginase‐1 depletion of essential T cell nutrients [4, 5, 6]. In addition, tumor‐derived receptor activator of nuclear factor‐κB ligand (RANKL) activates osteoclastogenesis through NF‐κB signaling, inducing pathologic bone resorption. This process liberates bone matrix growth factors (e.g., TGF‐β, IGF‐1), further fueling tumor proliferation and metastatic progression, thereby resulting in a self‐amplifying vicious cycle [7, 8, 9].

Notably, osteoclasts and immunosuppressive TAMs derive from monocytic precursors, with their differentiation governed by overlapping transcriptional regulatory genes [10, 11, 12]. Targeting these critical genes can simultaneously suppress osteoclastogenesis and TAM‐mediated immunosuppression, thereby inhibiting bone metastatic progression [13, 14, 15]. Transcriptomic profiling of monocyte‐derived lineages identifies triggering receptor expressed on myeloid cells 2 (TREM2) as a master regulator regulates osteoclastogenesis via NFATc1‐dependent NF‐κB activation and drives immunosuppressive TAM polarization through activating SYK/CEBPα axis and the STAT3/RORγ pathway [16, 17, 18, 19]. Critically, these processes engage in a self‐reinforcing vicious cycle: osteoclast‐mediated bone resorption liberates matrix‐embedded TGF‐β, which expands immunosuppressive TAMs populations, and in turn TAM‐derived IL‐10 suppresses T cell infiltration [20, 21, 22]. This immunosuppression enables tumor cells to overexpress RANKL, coupling immune evasion with osteolytic progression in the metastatic niche [23, 24, 25]. Thus, this immunosuppressive‐osteolytic cascade orchestrated by TREM2 may forge a sanctuary for tumor cells to evade immune attack, leading to a consequential therapeutic dilemma.

Consistent with this notion, accumulating evidence suggests that this TREM2‐centered myeloid program not only sustains local immunosuppression but may also contribute to the adaptive resistance to immunotherapy observed in bone‐metastatic TNBC [26, 27, 28]. In this setting, TREM2^+^ TAMs engage in reciprocal crosstalk with tumor cells by secreting IL‐10, TGF‐β, and arginase‐1 to impair effector T‐cell function, while concomitantly promoting the upregulation of immune‐evasive molecules such as PD‐L1 on tumor cells, thereby compromising the efficacy of immune checkpoint blockade [17, 27, 29]. Together, these findings support the notion that the immunosuppressive–osteolytic cascade driven by TREM2 is closely associated with compensatory resistance pathways.

Despite its compelling therapeutic promise, clinical translation of TREM2 inhibition in TNBC bone metastasis has remained elusive due to three mutually constraining barriers. First, the extreme genomic instability and intratumoral heterogeneity of TNBC preclude the use of predefined tumor receptors for reliable targeting [30, 31, 32]. Second, the dense mineralized bone matrix and acidic tumor microenvironment (TME) severely limit the penetration and stability of molecular inhibitors [33, 34, 35]. Third, systemic inhibition of myeloid programs carries a high risk of off‐target immune perturbation, a problem that is further exacerbated by the highly complex multicellular architecture of the TNBC bone metastatic niche [23, 36]. This niche is composed of osteolineage cells, immune cells, stromal cells, and endothelial cells that engage in extensive paracrine signaling and direct cell–cell interactions, rendering precise targeting of immunosuppressive macrophage subsets and efficient drug delivery exceptionally difficult [36]. Consequently, no available platform has yet been able to simultaneously overcome tumor heterogeneity, achieve myeloid‐selective gene silencing, and restrict therapeutic activity to bone metastases.

Small peptides offer superior tissue penetration compared with antibodies and large ligands and thus represent attractive candidates for navigating the physically shielded bone metastatic microenvironment [37, 38, 39]. However, most reported peptide‐ or ligand‐based nanocarriers rely on predefined tumor receptors, which are frequently absent or heterogeneously expressed in TNBC, resulting in poor targeting fidelity [40, 41, 42, 43]. To overcome this limitation, we employed phage display to enable unbiased, de novo identification of tumor‐homing peptides without presupposing receptor expression [44, 45, 46, 47]. Beyond tumor localization, selective modulation of myeloid subsets is essential for effective TREM2 silencing. Engineered outer membrane vesicles (OMVs) from hypoendotoxic *Escherichia coli* BL21 (ΔmsbB) intrinsically favor monocyte/macrophage uptake via Toll‐like receptor 4 (TLR4), offering a natural advantage over synthetic nanoparticles for myeloid targeting [48, 49, 50]. Nevertheless, existing OMV‐based systems lack tumor‐homing capacity and are predominantly sequestered by the liver and spleen following systemic administration, thereby failing to access bone metastatic lesions and risking off‐target immune activation [50, 51, 52]. To introduce precise spatial control, we incorporated a pH‐responsive poly(2‐ethyl‐2‐oxazoline) (PEOz) shield that masks OMVs under physiological conditions but dissociates within the acidic TME, thereby confining myeloid modulation to metastatic niches [53, 54].

**SCHEME 1:**
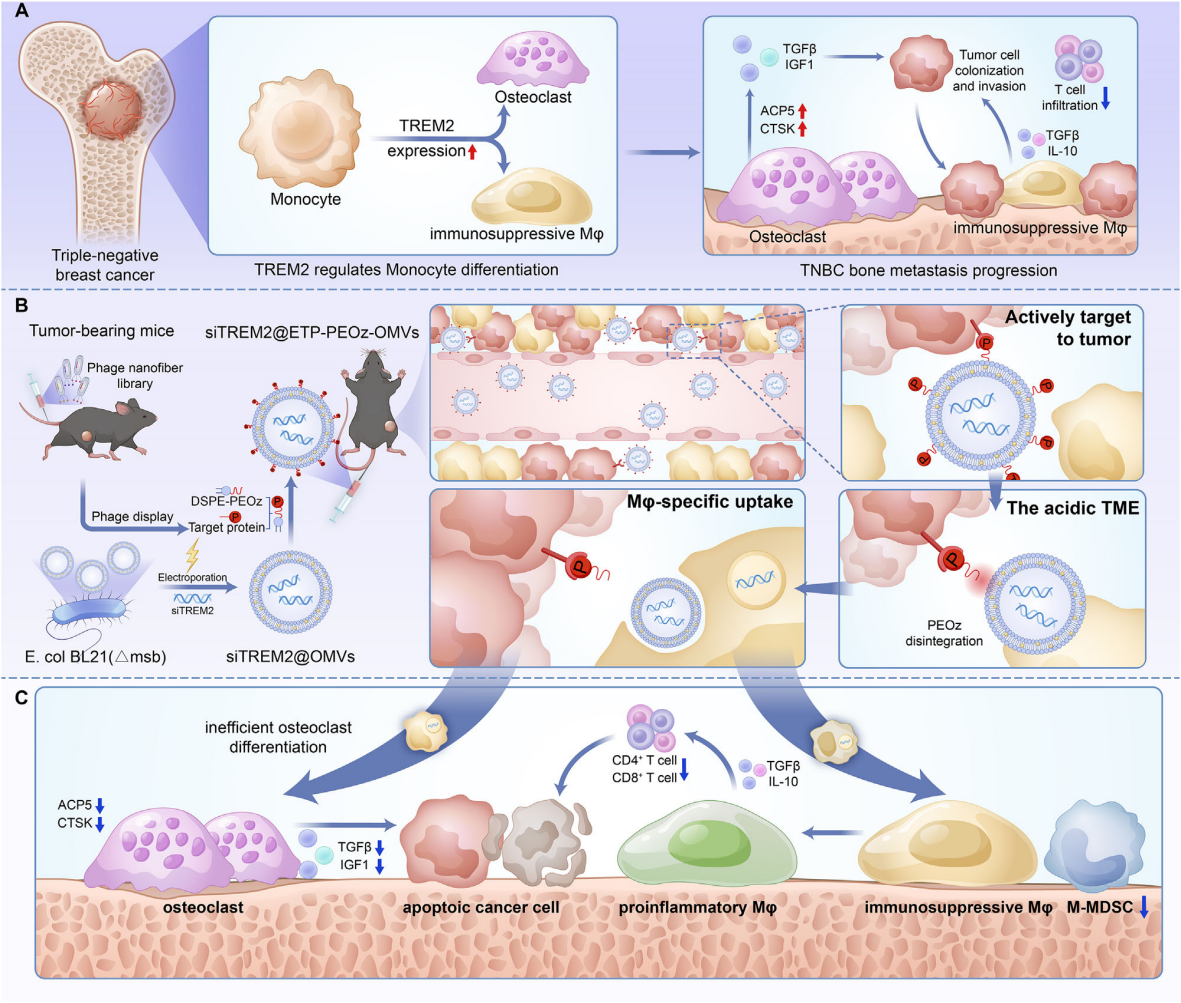
Schematic diagram of hierarchical targeting strategy against TNBC bone metastasis, designed to disrupt the osteolytic–immunosuppressive crosstalk. (A) In the microenvironment of TNBC bone metastasis, TREM2 drives the pathological differentiation of monocytes: promoting their polarization into immunosuppressive macrophages and differentiation into osteoclasts, thereby coupling immune evasion with osteolytic progression in the metastatic niche. (B) Preparation and the hierarchical tumor‐targeting process of therapeutic nanoplatform (siTREM2@ETP‐PEOz‐OMVs). (C) siTREM2@ETP‐PEOz‐OMVs precisely targets monocytes/macrophages in bone metastatic lesions, dually regulating their differentiation fates: reprogramming macrophages to alleviate the immunosuppressive phenotype while suppressing osteoclast differentiation, ultimately remodeling the antitumor immune microenvironment.

In consideration of the above, we developed a hierarchical targeting nanoplatform (siTREM2@ETP‐PEOz‐OMVs) that integrates phage display‐identified TNBC‐homing peptide ETP (E0771 targeting protein, with the sequence of GTNWSIHENNM) with myeloid‐selective OMVs. ETP was conjugated to DSPE‐PEOz on siTREM2‐loaded OMVs, enabling acid‐triggered exposure in metastatic niches for precise tumor binding and OMV release. This sequential targeting strategy achieves efficient TREM2 silencing in bone‐infiltrating myeloid cells, repolarizes TAMs toward proinflammatory phenotypes, enhances CD4^+^ and CD8^+^ T‐cell infiltration, and suppresses osteoclastogenesis, thereby dismantling the immunosuppressive–osteolytic cascade that drives TNBC bone metastasis. Our work establishes a translatable paradigm for overcoming tumor heterogeneity and immune resistance in target‐deficient metastatic cancers (Scheme 1).

## 2 Results

### 2.1 TREM2 Regulates Macrophage Immunosuppression and Osteoclast Differentiation to Promote TNBC Bone Metastasis

**FIGURE 1:**
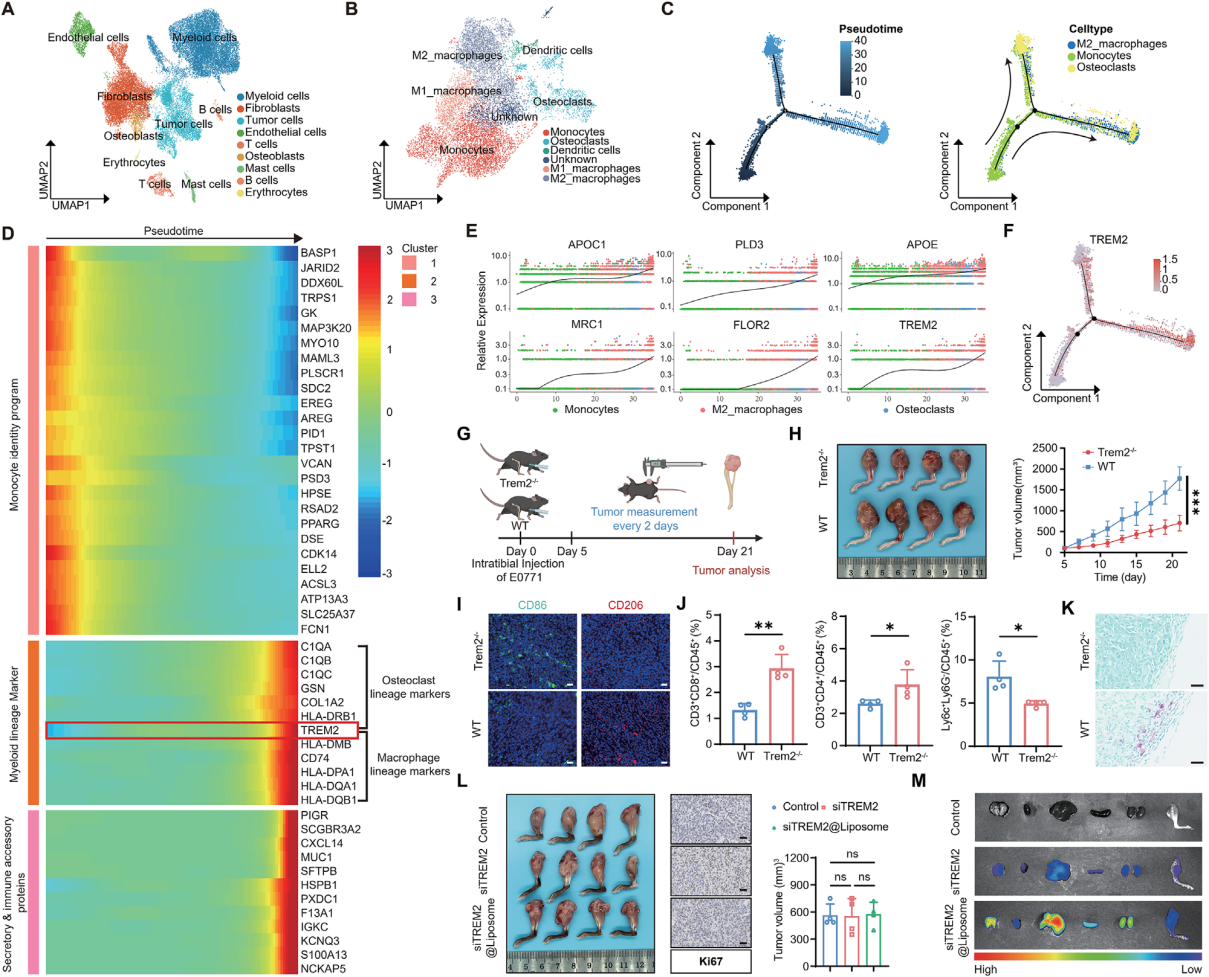
TREM2 regulates macrophage immunosuppression and osteoclast differentiation to promote TNBC bone metastasis. (A) UMAP plots of scRNA‐seq analysis showing cell type clusters in the TNBC bone metastasis lesions. (B) UMAP plots of myeloid cell subclusters. (C) Monocle trajectory of monocytes, M2_macrophages and osteoclasts, displaying pseudotime (left) and cell types (right). (D) The utilization of pseudotime analysis resulted in the production of a heat map that visually represented the alterations in gene expression pertaining to the top 50 genes associated with cellular fate throughout the pseudotime continuum. (E) TREM2 and coregulated gene expression profiles of monocytes with respect to pseudotime along the trajectory as inferred by Monocle2. The color of each point represents different cell types, and the horizontal axis represents time progression from left to right. (F) Cell trajectory projections of TREM2 level. (G) Schematic flow chart of TREM2^−/−^ and wild type TNBC orthotopic allografts model. (H) Representative images and tumor growth curve of primary tumors (*n* = 4 biologically independent experiments). (I) Immunofluorescence staining of CD86 and CD206 in tumor sections. Scale bars: 100 µm. (J) Flow cytometry analysis of tumor‐infiltrating CD8^+^ T cells, CD4^+^ T cells, and Ly6c^+^Ly6G^−^ M‐MDSCs (*n* = 4 biologically independent experiments). Gating strategies as in Figure S6A–C. (K) Representative TRAP staining images of tumor–bone interface sections from WT and Trem2^−/−^ mice, showing reduced TRAP‐positive osteoclasts (red) in Trem2^−/−^ group. Scale bars: 25 µm. (L) Representative photographs of excised tumor masses and Ki67 immunohistochemical staining (brown nuclei indicate proliferating cells) from groups treated with saline, naked siTREM2, or siTREM2@Liposome on Day 21. Bar graph shows quantified tumor volumes (mm^3^) for each group (*n* = 4 biologically independent experiments). Scale bars: 10 µm. (M) Biodistribution of Cy5‐siTREM2 and Cy5‐siTREM2@liposome in heart, liver, spleen, lung, kidney, and tumor after 6 h i.v. injection. Data were presented as the mean ± standard deviation (SD) (unpaired two‐tailed Student's *t*‐test, ns, no significance. *p < 0.05, **p < 0.01, ***p < 0.001).

To systematically characterize the cellular landscape of TNBC bone metastasis, we conducted unbiased single‐cell RNA sequencing (scRNA‐seq) derived from human TNBC bone metastasis lesions. We performed cell classification and marker gene identification using scanpy. Based on the expressions of canonical markers, cells were divided into 10 major cell types, including epithelial cells, fibroblasts, endothelial cells, myeloid cells, T cells, B cells, neutrophils, NK cells, pericytes, and mast cells (Figure 1A). Given the critical role of myeloid cells in the TME and bone metastasis, we next aimed to identify and define distinct subpopulations within the myeloid compartment. Unsupervised subclustering of myeloid cells revealed five distinct populations: M1‐macrophages (TNF, CCL3, and CCL20), M2‐macrophages (Mrc1, TREM2, and FOLR2), osteoclasts (ACP5, CTSK, and NFATC1), dendritic cells (FCER1A and CD1C), and monocytes (VCAN, ANPEP, and CD300E) (Figure 1B and Figure S1).

Based on the important role of osteoclasts and immunosuppressive macrophages in TNBC bone metastasis, both originating from the monocyte–macrophage lineage, we performed cell trajectory analysis by using the monocyte, M2_macrophage (a subset known to be immunosuppressive) and osteoclast clusters as input cells to trace the potential differentiation trajectories. The inferred differentiation trajectories are presented in Figure 1C. To identify key genes dynamically regulated along this monocyte differentiation path, we analyzed differentially expressed genes (DEGs) across pseudotime and categorized them into three clusters: Cluster 1 (down‐regulated along pseudotime) and Clusters 2 and 3 (up‐regulated along pseudotime). Cluster 2 primarily includes genes associated with myeloid lineage differentiation, encompassing both osteoclast and macrophage lineage markers (Figure 1D). Notably, TREM2 emerged as a shared gene associated with both the macrophage and osteoclast lineages within Cluster 2. Its expression was markedly upregulated starting from early stages of differentiation and exhibited a significant increase toward terminal states. suggesting its potential role as a hub gene common to both differentiation branches. Further analysis of pseudotemporal dynamics revealed significantly elevated expression curves of TREM2 and its coregulated genes (APOC1, PLD3, APOE, MRC1, FOLR2) (Figure 1E). Expression analysis across the two inferred differentiation branches (toward osteoclasts and M2‐macrophages) also demonstrated a progressive increase in TREM2 expression along both trajectories (Figure 1F). Therefore, we speculate that TREM2 may play a key regulatory role involving the differentiation of monocytes into osteoclasts and immunosuppressive macrophages in TNBC bone metastasis.

To validate whether TREM2 regulates the differentiation of monocytes into osteoclasts and M2 macrophages, thus promoting TNBC bone metastasis, we established an intratibial TNBC bone metastasis model in TREM2 knockout (Trem2^−/−^) and wild‐type (WT) mice (Figure 1G). As shown in Figure 1H and Figure S2, TREM2 deficiency significantly inhibited tumor growth in TNBC bone metastatic lesions compared to WT controls. Subsequent comprehensive immune profiling demonstrated profound alterations in the TME: immunofluorescence and flow cytometry analyses showed increased infiltration of iNOS, CD86^+^, CD4^+^, and CD8^+^ cells alongside decreased Arg1, CD206^+^ populations in Trem2^−/−^ mice (Figure 1I and Figures S3–S5). Quantitative assessment revealed a 2.7‐fold increase in iNOS^+^F4/80^+^ macrophages (increased from 3.33% to 9.07%) and a 58% reduction in Arg1^+^F4/80^+^ macrophages (decreased from 12.85% to 5.3%) (Figure S4). Notably, we observed a 123% increase in cytotoxic CD3^+^CD8^+^ T cells and 46% increase in CD3^+^CD4^+^ T cells, coupled with a 39% reduction in M‐MDSCs (monocytic MDSCs) in Trem2^−/−^ mice (Figure 1J). The gating strategy is shown in Figure S6. In addition, histological tartrate‐resistant acid phosphatase (TRAP) staining was performed to assess osteoclast formation in the tumor region. Compared to control mice, Trem2^−/−^ mice showed a significant reduction in TRAP‐positive cells at the tumor–bone interface of the tibia, indicating that Trem2 gene knockout markedly inhibits osteoclast formation in vivo (Figure 1K). These findings suggest that TREM2 is a potential therapeutic target for TNBC bone metastasis. Its knockout promotes macrophage polarization toward a proinflammatory phenotype and enhances T cell infiltration while suppressing osteoclast‐mediated bone destruction and tumor invasion, thereby effectively reshaping the tumor immune microenvironment.

To translate our genetic findings into a therapeutic strategy, we evaluated both naked siTREM2 and liposome‐encapsulated siTREM2 (siTREM2@Liposome) in a murine intratibial TNBC model. However, neither treatment significantly inhibited tumor growth or altered the immune microenvironment by day 21 compared to controls (Figure 1L and Figure S7). Biodistribution detection revealed poor tumor accumulation, with both Cy5.5‐siTREM2 and Cy5.5‐siTREM2@Liposome predominantly localizing to the liver and showing minimal retention at the bone metastatic site (Figure 1M). These results mirror the limitations of conventional chemotherapy in tumor metastases and highlight two critical barriers: inefficient drug delivery to TNBC bone lesions due to their lack of specific molecular targets, and the inability of current systems to precisely target and modulate TREM2 expression in monocytes/macrophages within the bone microenvironment. Overcoming these challenges require the development of advanced delivery platforms capable of tumor‐specific homing and monocyte‐selective gene regulation to disrupt the vicious cycle of osteoclast, M2 macrophage, and tumor cell crosstalk in TNBC bone metastasis.

### 2.2 Preparation and Characterization of siTREM2@ETP‐PEOz‐OMVs

**FIGURE 2:**
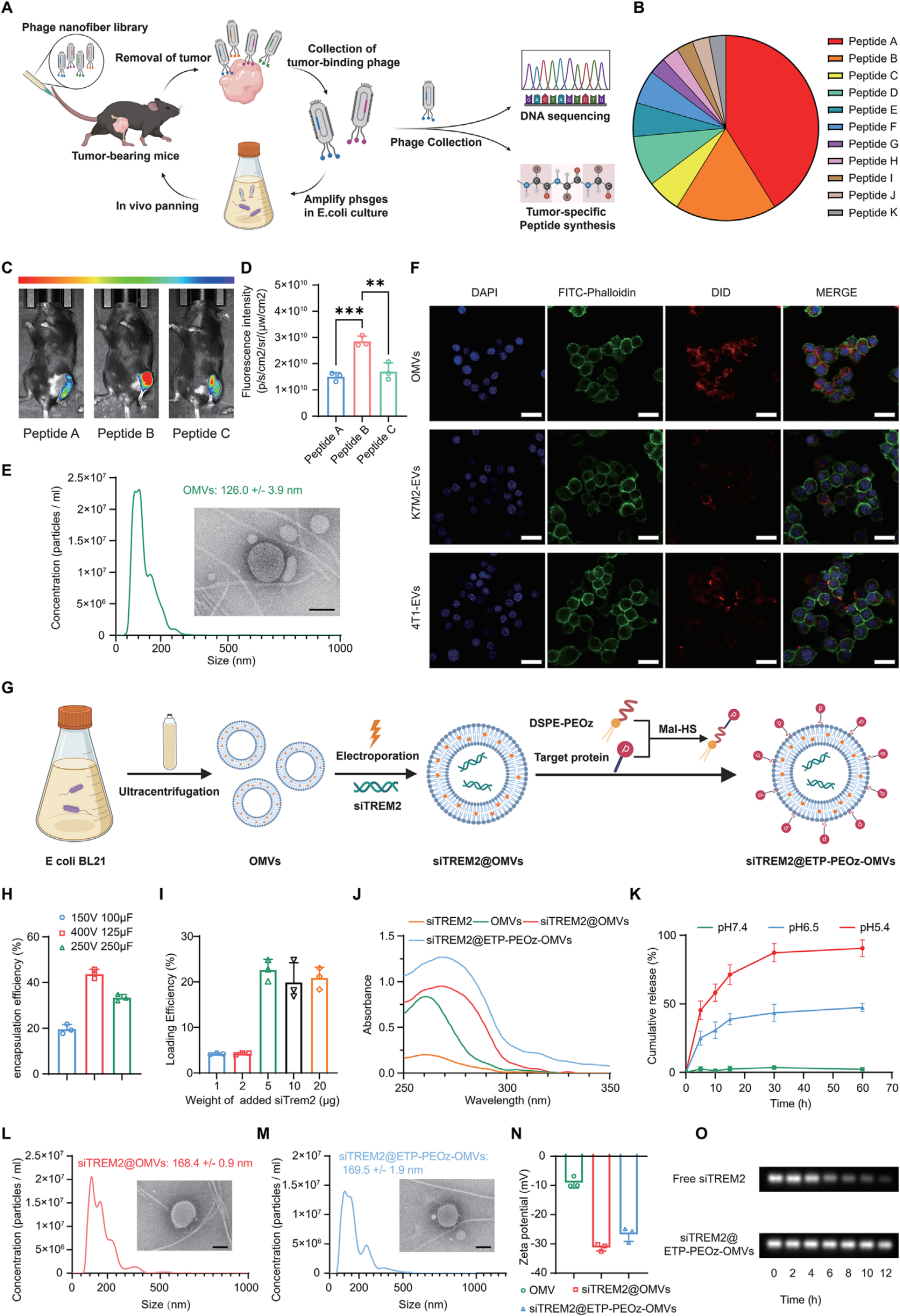
Preparation and characterization of siTREM2@ETP‐PEOz‐OMVs. (A) Schematic description of in vivo phage display biopanning for selecting TNBC‐targeting peptides from a phage library. (B) Summary of peptide frequencies in each round of phage display against E0771 tumors. (C) In vivo living imaging for detection of specific accumulation of tumor homing peptides at the E0771 tumors. (D) Quantitative analysis of peptide homing efficiency to tumor sites (*n* = 3 biologically independent experiments). (E) TEM images (right) showing bowl‐like bilayer structure of empty OMVs and NTA histograms (left) of particle size distribution (average diameter: 126 ± 3.9 nm). Scale bars: 100 nm. (F) Representative confocal fluorescence images of RAW264.7 cells incubated with Did‐labeled OMVs, K7M2‐EVs, or 4T1‐EVs for 1 h, stained with DAPI (blue) and FITC‐phalloidin (green). Red fluorescence indicates vesicle uptake, with higher intensity in OMVs group. Scale bars: 20 µm. (G) Schematic illustration of the preparation process of siTREM2@ETP‐PEOz‐OMVs. (H,I) Loading efficiency and encapsulation efficiency of siTREM2 in OMVs. (J) UV–vis absorbance spectra. (K) Kinetic release profiles of siTREM2‐loaded OMVs from siTREM2@ETP‐PEOz‐OMVs in phosphate‐buffered saline (PBS) at pH 7.4 (physiological) and pH 5.4 (acidic TME simulation), measured by fluorescence over 60 min. Data show ∼90% release at pH 5.4 within 30 min. (L,M) TEM images and NTA results of siTREM2@OMVs and siTREM2@ETP‐PEOz‐OMVs. Scale bars: 100 nm. (N) Zeta potential analyses of OMVs, siTREM2@OMVs and siTREM2@ETP‐PEOz‐OMVs (*n* = 3 biologically independent experiments). (O) Electrophoresis strips of free siTREM2 and siTREM2@ETP‐PEOz‐OMVs after incubation with FBS at different time points (0–12 h). Data were presented as the mean ± SD (one‐way analysis of variance (ANOVA) comparisons tests, **p < 0.01, ***p < 0.001).

To precisely regulate TREM2 expression in monocytes/macrophages in TNBC bone metastases, we developed a hierarchical targeted therapy system that integrates tumor‐specific localization with gene‐selective modulation for effective TREM2 targeting. Through three rounds of in vivo phage screening against TNBC bone metastatic lesions (Figure 2A), we identified two high‐affinity peptides (TIPNLTRVSNIV, peptide A; GTNWSIHENNM, peptide B) with a higher recurrence frequency (Table S1 and Figure 2B). To enable imaging and conjugation, a Gly‐Gly‐Gly‐Ser‐Cys linker was incorporated at the C‐terminal of both peptides, with mass spectrometry and chromatogram confirming the successful synthesis of Peptide A and Peptide B (Figure 2C and Figure S8). To evaluate the tumor‐targeting capability of the selected peptides, we performed in vivo fluorescence imaging using Cy5.5‐labeled peptides, with SWSNASDYHIGA (peptide C) serving as a nonspecific control. Notably, peptide B exhibited significantly stronger accumulation at TNBC bone metastasis sites compared to peptides A and C (Figure 2C), with quantitative analysis revealing approximately twofold higher fluorescence intensity for peptide B (Figure 2D). In vitro fluorescence staining revealed the superior affinity of peptide B for E0771 cells, exhibiting twofold higher Cy5.5 signals compared to peptides A and C (Figure S9), consistent with its in vivo targeting performance. Based on these findings, we designated peptide B as ETP, which demonstrates exceptional potential of this phage display screened peptide for targeting highly heterogeneous TNBC tumors through its validated in vivo homing capability and in vitro binding specificity.

Next, we evaluated characteristics of OMVs. Morphologically, transmission electronic microscopy (TEM) imaging of OMVs displayed a bowl‐like bilayer structure and nanoparticle tracking analysis (NTA) showed that the average diameters of the empty OMVs was 126 ± 3.9 nm (Figure 2E). In order to identify the affinity of OMVs phagocytized by monocytes/macrophages, Did‐labelled OMVs, K7M2 EVs, and 4T1 EVs were incubated with RAW264.7 cells, respectively (Figure 2F). Confocal images showed significantly higher fluorescence intensity of OMVs in RAW264.7 cells compared to K7M2 EVs and 4T1 EVs. Further specificity testing across multiple cell lines (E0771 cells, RAW264.7 cells, and 3T3‐L1 preadipocytes) confirmed that OMVs were predominantly internalized by RAW264.7 cells, with minimal uptake in E0771 cells and 3T3‐L1 cells (Figure S10). These results establish OMVs as an ideal delivery platform for monocytes/macrophages‐specific gene regulation in the TNBC bone metastatic microenvironment.

The hierarchical nanoplatform was constructed through a multistep assembly process (Figure 2G). Initial siTREM2 loading into OMVs was achieved via electroporation, yielding an encapsulation efficiency (EE) of 43.75 ± 2.03% at optimal parameters (400 V, 125µF), with loading saturation occurring at a 1:2 mass ratio of siTREM2: OMVs and the corresponding loading efficiency (LE) of 22.60 ± 2.35% (Figure 2H,I). To achieve acidic TME‐triggered prerelease of siTREM2@OMVs, the triblock polymer DSPE‐PEOz‐ETP was designed (Figure S11). The successful synthesis of DSPE‐PEOz‐ETP was characterized by ^1^H NMR (Figure S12). To evaluate the optimized ligand modification ratio, DSPE‐PEOz‐ETP: OMV protein mass ratios of (1:20 to 1:1) were used to generate ETP‐PEOz‐OMVs and the degree of modification in OMVs was saturated after incubation ratios equal to or greater than 1:5 (Figure S13). Also, siTREM2@ETP‐PEOz‐OMVs exhibited the characteristic absorption peaks of siTREM2 (approximately 260 nm) in the UV–vis absorbance spectra, indicating successfully achieved siRNA loading (Figure 2J). To confirm the pH‐responsive cleavage of the DSPE‐PEOz‐ETP conjugate, FTIR spectroscopy was performed before and after acid exposure (pH 5.4, 30 min). The spectra revealed a marked reduction in the amide C═O peak at ∼1640 cm^−^^1^ (corresponding to the amide C═O stretch in the PEOz backbone) and the appearance of an O─H stretch at ∼3400 cm^−^^1^ (indicative of O─H stretching from hydrolysis products), verifying acid‐triggered hydrolysis of the pH‐sensitive amide bonds in PEOz and leading to the dissociation of the polymer shield and subsequent release of the ETP‐conjugated OMVs (Figure S14). This mechanism underpins the controlled release behavior observed in functional assays: as shown in Figure 2K, siTREM2@ETP‐PEOz‐OMVs achieved approximately 90% release of siTREM2‐loaded OMVs within 30 min under simulated acidic TME conditions (pH 5.4) [55], whereas no significant OMVs release occurred at physiological pH (pH 7.4), suggesting its stable property under physiological conditions and ideal pH‐responsive performance in TME.

TEM imaging further revealed that both siTREM2@OMVs and siTREM2@ETP‐PEOz‐OMVs maintained the characteristic bowl‐shaped bilayer structure of native OMVs, with systematic size increases to 168.4 ± 0.9 nm (siTREM2@OMVs) and 169.5 ± 1.9 nm (siTREM2@ETP‐PEOz‐OMVs) (Figure 2L,M), indicating the uniform size distributions and satisfactory size stability. Moreover, the Zeta potential decreased from OMVs (−8.96 ± 1.88 mV) to siTREM2@OMVs (−31.17 ± 1.26 mV) and siTREM2@ETP‐PEOz‐OMVs (−26.7 ± 2.45 mV were all negative, consistent with previously reported zeta potential values of cell membranes or conventional liposomes [54, 56] (Figure 2N). As shown in Figure 2O, siTREM2@ETP‐PEOz‐OMVs retained detectable electrophoretic strips after 12 h incubation, in stark contrast to the nearly complete degradation of free siTREM2, demonstrating that OMV encapsulation effectively shields siRNA from serum nuclease‐mediated degradation. Furthermore, CCK‐8 assays demonstrated high biocompatibility of both empty OMVs and siTREM2@ETP‐PEOz‐OMVs in RAW264.7 cells, with approximately 91% viability at 1 µg/mL, confirming the intrinsic biosafety of this system (Figure S15).

### 2.3 Hierarchical Targeting of TNBC Bone Metastasis and Monocytes/Macrophages in TME by siTREM2@ETP‐PEOz‐OMVs

**FIGURE 3:**
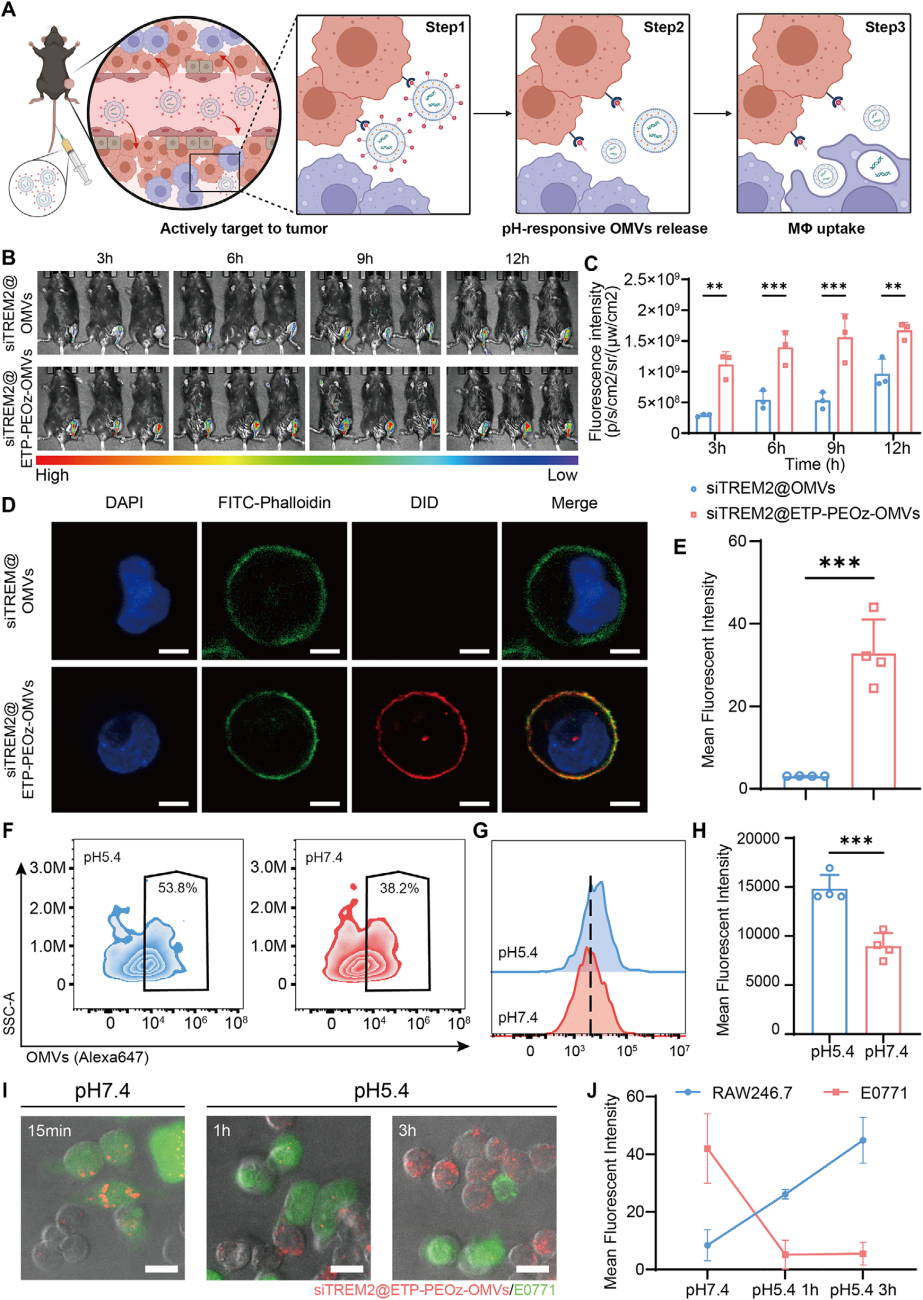
Hierarchical targeting of TNBC bone metastasis lesions and macrophages in TME by siTREM2@ETP‐PEOz‐OMVs. (A) Schematic illustration of the sequential targeting mechanism. (B) In vivo bioluminescence images of E0771 tumor‐bearing mice at 3, 6, 9, and 12 h postinjection of Did‐labeled siTREM2@OMVs or siTREM2@ETP‐PEOz‐OMVs, showing enhanced tumor accumulation in the targeted group. (C) Fluorescence intensity of siTREM2@ OMVs and siTREM2@ETP‐PEOz‐OMVs in the tumor site (*n* = 3 biologically independent experiments). (D) Confocal fluorescence images of E0771 cells incubated with Did‐labeled siTREM2@OMVs or siTREM2@ETP‐PEOz‐OMVs (50 ng/mL, 60 min), stained with DAPI (blue) and FITC‐phalloidin (green). Red fluorescence indicates nanoparticle binding, with ∼tenfold higher intensity in targeted group. Scale bars: 5 µm (*n* = 4 biologically independent experiments). (E) Fluorescence intensity of siTREM2@ETP‐PEOz‐OMVs versus siTREM2@ OMVs incubated with E0771 cells (*n* = 4 biologically independent experiments). (F–H) Flow cytometry plots (F), histograms (G), and quantification (H) of Did‐positive RAW264.7 cells after incubation with siTREM2@ETP‐PEOz‐OMVs at pH 7.4 vs. pH 5.4, showing increased phagocytosis rate (from 38.2% to 53.8%) and mean fluorescence intensity (65% rise) at acidic pH. (I) Time‐lapse confocal images of cocultured E0771 (green, CFSE) and RAW264.7 cells with Did‐labeled siTREM2@ETP‐PEOz‐OMVs (red) at pH 7.4 (initial binding to tumor cells) and after pH drop to 5.4 (OMV release and macrophage uptake over 1–3 h). Scale bar = 10 µm. (J) Quantitative analysis of the fluorescence intensity of E0771 cells and RAW264.7 cells in the different pH (*n* = 3 biologically independent experiments). Data were presented as the mean ± SD (one‐way ANOVA comparisons tests, **p < 0.01, ***p < 0.001).

Based on our design, siTREM2@ETP‐PEOz‐OMVs firstly aggregated at the site of TNBC bone metastases via blood circulation after injection. Upon acidic TME‐triggered disassembly, the released OMVs are specifically phagocytosed by monocytes/macrophages thus siTREM2 is delivered to exert silencing effects (Figure 3A). To further explore the tumor tropism of siTREM2@ETP‐PEOz‐OMVs in vivo, Did‐labeled siTREM2@OMVs and siTREM2@ETP‐PEOz‐OMVs were injected intravenously into the tibial E0771 bone metastasis mice model. As shown in Figure 3B,C, Did‐labeled siTREM2@ETP‐PEOz‐OMVs exhibited rapid tumor accumulation within 3 h postinjection, with fluorescence intensity doubling that of nontargeted siTREM2@OMVs. To quantitatively characterize the systemic pharmacokinetic behavior underlying this efficient tumor accumulation, we determined the blood circulation profiles. The siTREM2@ETP‐PEOz‐OMVs exhibited a significantly prolonged circulation half‐life (*t*_1/2_ = 1.851 h) compared to the nontargeted siTREM2@OMVs (*t*_1/2_ = 1.036 h) (Figure S16). This enhancement is attributed to the hydrophilic shielding effect of the DSPE‐PEOz2k coating, which reduces nonspecific protein adsorption and delays clearance by the mononuclear phagocyte system, thereby allowing more nanoparticles to reach the tumor site over time. Notably, this prolonged circulation contributed to superior and persistent tumor retention. The tumor‐specific fluorescence intensity of siTREM2@ETP‐PEOz‐OMVs was 1.6‐fold higher than the control at 24 h and further increased to 2.0‐fold at 72 h (Figure S17), confirming the enhanced tumor tropism and retention conferred by our targeted design. Moreover, to investigate the cancer targeting capability of siTREM2@ETP‐PEOz‐OMVs in vitro, E0771 cells were observed under a confocal microscope after interacted with siTREM2@OMVs or siTREM2@ETP‐PEOz‐OMVs (Figure 3D). Quantitative analysis showed that siTREM2@ETP‐PEOz‐OMVs exhibited approximately tenfold higher binding affinity to E0771 cells compared to unmodified siTREM2@OMVs, further validating ETP‐mediated tumor targeting (Figure 3E).

To further validate the effect of DSPE‐PEOz in improving the delivery efficiency of siTREM2 into monocytes/macrophages, we established a coculture system of RAW 264.7 cells and E0771 tumor cells to simulate the TNBC bone metastatic microenvironment. Flow cytometry of Did‐labeled siTREM2@ETP‐PEOz‐OMVs revealed significantly enhanced uptake under acidic conditions (pH 5.4), with phagocytosis rates increasing from 38.2% to 53.8% and mean fluorescence intensity rising by 65% compared to pH7.4 (Figure 3F–H). These results further demonstrate that the pH‑responsive design facilitates rapid OMV release at the acidic TNBC metastatic site and their subsequent internalization. Representative confocal image showed the complete hierarchical targeting delivery process (Figure 3I). At pH 7.4, siTREM2@ETP‐PEOz‐OMV binds firstly to the surface of E0771 cells within 15 min due to ETP's tumor affinity. When the pH dropped to 5.4, PEOz disintegrated, and the released siTREM2@OMVs were gradually taken up by macrophages. Quantitative analysis confirmed that this uptake was progressively enhanced over time (Figure 3J).

To further validate macrophage‐preferential uptake in a physiologically relevant system, we examined OMV internalization and its relationship with TLR4 expression across the differentiation stages of primary human monocyte‐derived macrophages (hMDMs). Flow cytometry analysis revealed that TLR4 surface expression progressively increased from freshly isolated monocytes (5.70%) to mature macrophages (77.10%) and remained elevated in M2 macrophages (76.43%). This increase was accompanied by the corresponding enhancement in OMV internalization, rising from 4.46% in monocytes to 87.57% in mature macrophages and remaining high in M2 macrophages (85.77%) (Figure S18). To further determine whether TLR4 signaling mediates OMV uptake, cells were pretreated with the TLR4 inhibitor TAK‐242 prior to OMV exposure. TLR4 blockade significantly reduced OMV internalization by approximately 55% in monocytes and 59% in macrophages (Figure S19), indicating that OMV uptake is largely dependent on TLR4 signaling.

Importantly, we next sought to determine whether such macrophage‐preferential uptake also occurs in vivo. Multicolor flow cytometry analysis of enzymatically digested tibial tumor tissues from E0771 bone metastasis–bearing mice 24 h after intravenous administration of Dio‐labeled siTREM2@ETP‐PEOz‐OMVs revealed that OMV uptake was highly enriched in the myeloid compartment, including CD11b^+^ monocytes and F4/80^+^ macrophages, whereas T cells and CD45^−^ nonimmune cells exhibited markedly lower OMV^+^ fractions (Figure S20). These results provide direct in vivo evidence at single‐cell resolution that our nanoplatform preferentially accumulates in tumor‐associated myeloid cells within the bone metastatic niche, supporting the proposed hierarchical targeting mechanism.

Finally, to delineate the intracellular trafficking of the delivered siRNA, Cy3‐labeled siTREM2 encapsulated in OMVs was tracked in RAW264.7 cells. Confocal imaging revealed that at 3 h postincubation, the Cy3‐siTREM2 signal (red) showed significant overlap with Lysotracker Green‐labeled lysosomes (green), confirming endolysosomal trafficking. By 6 h, the majority of the red siRNA signal had separated from the lysosomal compartments and was diffusely distributed in the cytoplasm, demonstrating efficient endolysosomal escape (Figure S21). This timely escape is critical for protecting siRNA from degradation and enabling effective target gene silencing. Collectively, these results demonstrate that siTREM2@ETP‐PEOz‐OMVs achieve hierarchical targeting of TNBC bone metastasis through sequential tumor recognition, TME‐responsive OMV release, and preferential delivery of siTREM2 to monocytes/macrophages.

### 2.4 siTREM2@OMVs Reprogrammed TAMs into Proinflammatory Phenotype and Enhanced Phagocytosis of Cancer Cells

**FIGURE 4:**
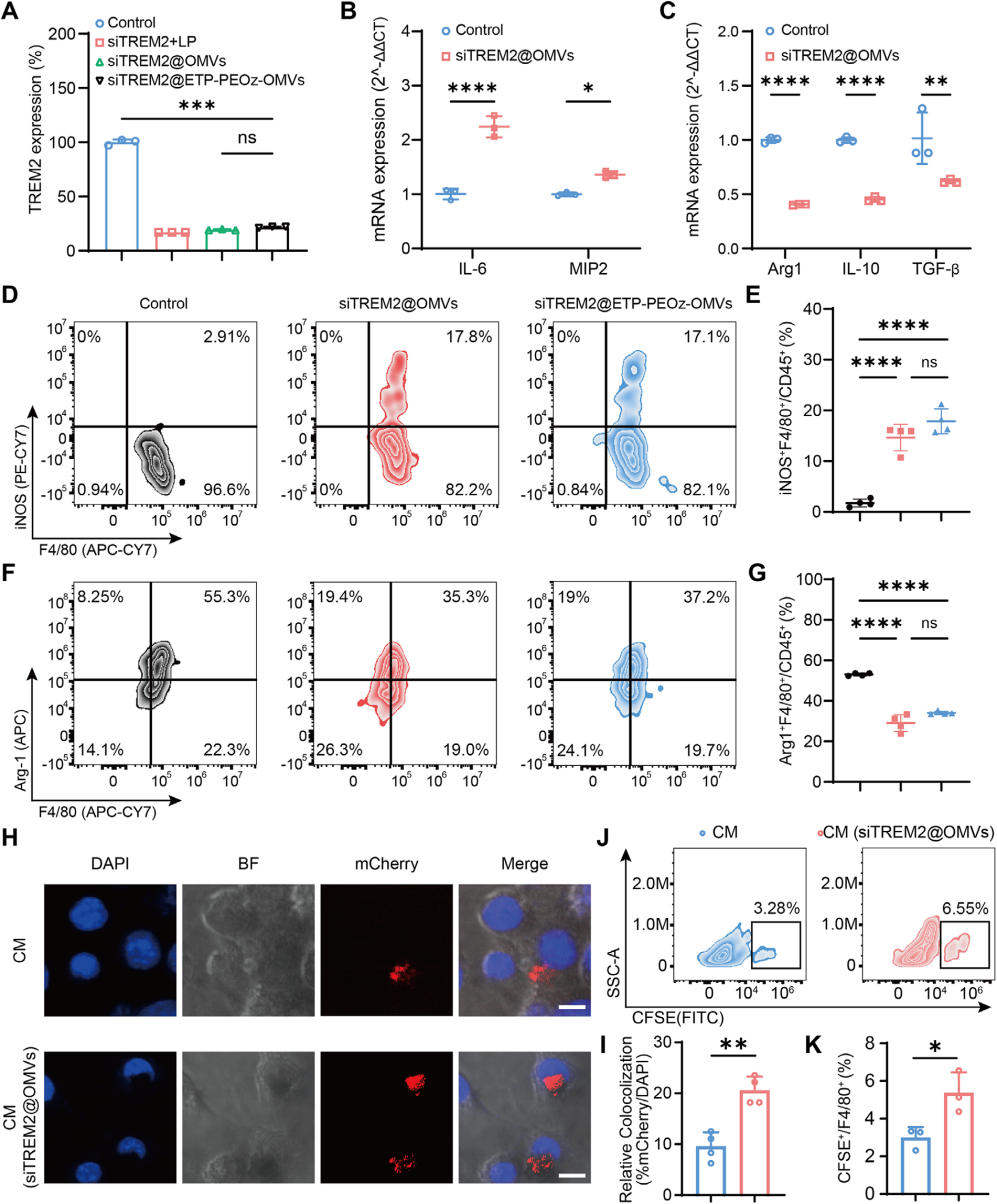
siTREM2@OMVs reprogrammed TAMs into proinflammatory phenotype and enhanced phagocytosis of cancer cells. (A) TREM2 expression of Raw 264.7 cells (pretreated with E0771 tumor conditioned medium for 24 h) after the indicated treatment detected by RT‐qPCR (*n* = 3 biologically independent experiments). (B,C) RT‐qPCR of the expression levels of IL6, MIP2, TGF‐β, IL10, and Arg1 after treated with siTREM2@OMVs (*n* = 3 biologically independent experiments). (D–G) Flow cytometry contour plots (D,F) and quantification (E,G) of iNOS+ (proinflammatory) and Arg1+ (immunosuppressive) macrophages in TAM‐like RAW264.7 cells (pretreated with E0771‐conditioned medium) after treatment with siTREM2@OMVs or siTREM2@ETP‐PEOz‐OMVs. Gating strategies as in Figure S6F. (H,I) Confocal images (H) of RAW264.7 cells (pretreated with E0771‐conditioned medium) phagocytosing mCherry‐labeled E0771 cells (red) after PBS or siTREM2@OMVs treatment (24 h). Scale bars: 10 µm (*n* = 4 biologically independent experiments). (J,K) Flow cytometry plots (J) and bar graph (K) of F4/80+ CFSE+ double‐positive RAW264.7 cells (phagocytic macrophages) after coculture with CFSE‐labeled E0771 cells, showing phagocytosis increase from 3.20% to 6.55% with siTREM2@OMVs (*n* = 4 biologically independent experiments). Gating strategies as in Figure S6G. Data were presented as the mean ± SD (one‐way ANOVA comparisons tests, ns, not significant, *p < 0.05, **p < 0.01, ***p < 0.001, ****p < 0.0001).

To evaluate the specific delivery and immunomodulatory effects of our nanoplatform, we used RAW264.7 cells (pretreated with E0771 tumor conditioned medium for 24 h) to mimic TAMs and cells were treated with siTREM2 plus transfection reagents (Lipofectamine 2000; siTREM2+LP), siTREM2@OMV or siTREM2@ETP‐PEOz‐OMV for 24 h, respectively. Real‐time polymerase chain reaction (RT‐qPCR) analysis demonstrated that compared with saline, different OMVs downregulated the mRNA expression level of TREM2, and this capability was comparable to that of the positive control Lipo2000 (Figure 4A). In addition, we found that treated macrophages further secreted proinflammatory cytokines such as interleukin 6 (IL‐6) and macrophage inflammatory protein 2 (MIP2), which were increased by 124% and 36%, respectively, whereas the expression of immunosuppressive factors such as Arg1, IL‐10, and TGFβ was reduced by 59%, 55%, and 40% (Figure 4B,C). This suggests that after achieving TREM2 knockdown, the delivery system can regulate the macrophage phenotype to proinflammatory macrophages and reduce the secretion of immunosuppressive factors such as IL‐10, thereby weakening their effects on T cell tumor infiltration and function.

To further quantitatively assess macrophage polarization following TREM2 silencing, we performed flow cytometric analysis of bone marrow‐derived macrophages (BMDMs) pretreated with E0771 tumor‐conditioned medium. Compared to untreated controls, both siTREM2@OMVs and siTREM2@ETP‐PEOz‐OMVs treatment groups significantly remodels macrophage phenotypes: the proportion of proinflammatory iNOS^+^ macrophages increased dramatically from 2.91% to 17.8% and 17.1%, respectively (Figure 4D,E), while the immunosuppressive Arg1^+^ macrophage population decreased from 55.3% to 35.3% and 37.2% (Figure 4F,G), confirming the previous RT‐qPCR results. To further enhance clinical relevance and validate these findings in a human‐relevant system, we used primary hMDMs isolated from healthy donors. CD14^+^ monocytes were differentiated into mature macrophages with M‐CSF for 7 days and then polarized to M2 macrophages by stimulation with IL‐4 and IL‐13 for 24 h. Treatment with siTREM2@OMVs robustly repolarized these primary human macrophages toward a proinflammatory phenotype, increasing the proportion of CD86^+^ cells 3.6‐fold (from 6.6% to 23.9%) while decreasing CD206^+^ cells by 62% (from 21.1% to 8.1%) (Figure S22).

We also functionally validated the therapeutic potential of macrophage reprogramming by evaluating the phagocytic ability of siTREM2@OMV‐treated RAW264.7 cells toward mCherry‐labeled E0771 tumor cells [57, 58]. Significant whole‐cell phagocytosis was observed by confocal microscopy upon treatment with siTREM2@OMVs compared with control group (Figure 4I). Similar results were observed by flow cytometry of CFSE‐labeled tumor cells—where the proportion of phagocytically active (F4/80^+^CFSE^+^) macrophages increased from 3.20% to 6.55% following siTREM2@OMVs treatment (Figure 4J). These results confirmed that siTREM2@OMVs provided functional evidence for antitumor strategies against TNBC bone metastasis by enhancing phagocytosis of tumor cells by macrophages.

### 2.5 siTREM2@OMVs Suppressed the Osteoclastogenesis In Vitro

**FIGURE 5:**
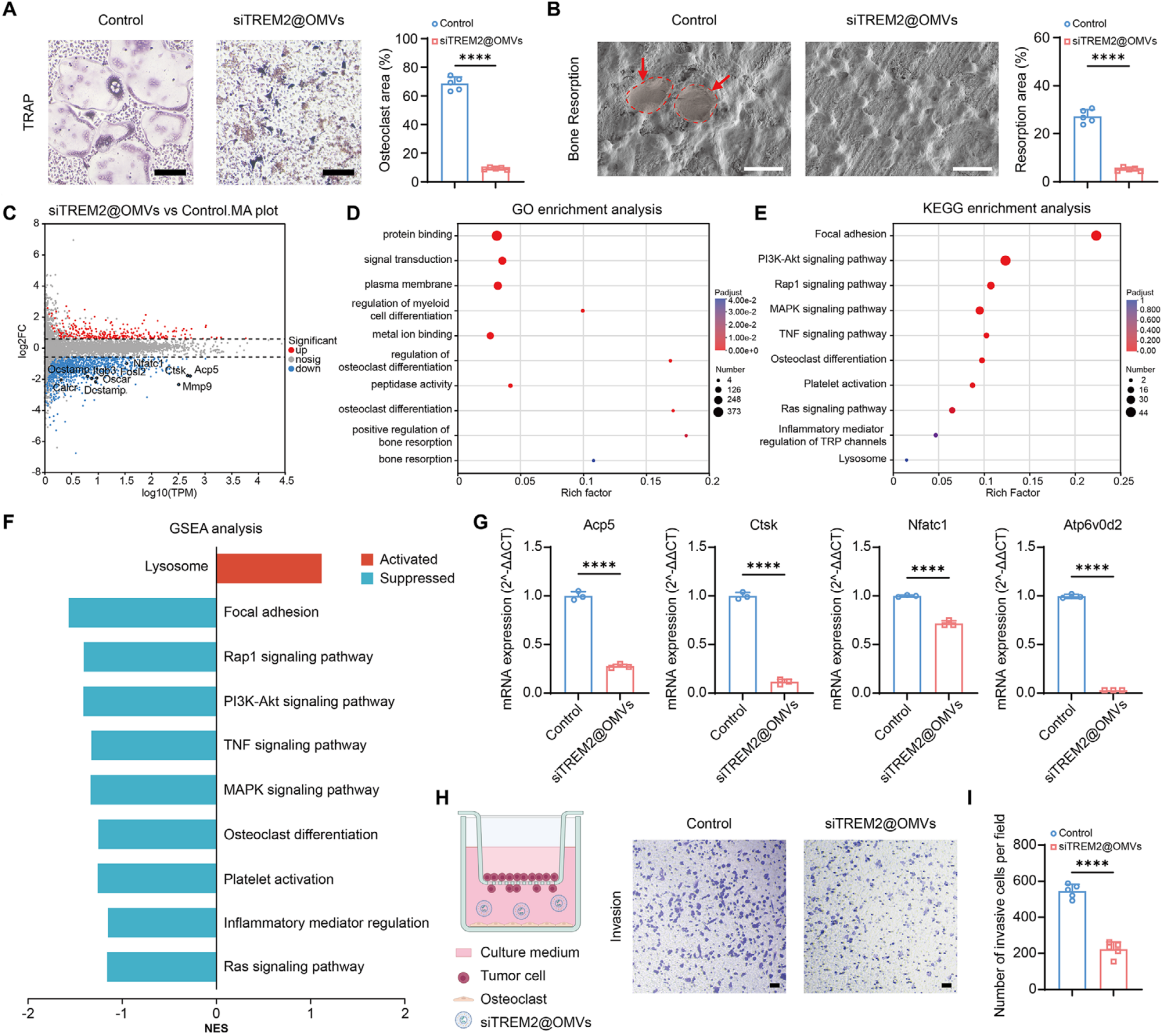
siTREM2@OMVs suppressed the osteoclastogenesis in vitro. (A) TRAP staining (left) of BMDMs induced to osteoclasts with M‐CSF/RANKL ± siTREM2@OMVs, showing reduced multinucleated TRAP^+^ cells (purple). Bar graph (right) quantifies osteoclast area (81% reduction; *n* = 5 biologically independent experiments). Scale bars: 200 µm. (B) SEM images (left) of bone resorption pits on bovine bone slices by induced osteoclasts ± siTREM2@OMVs. Bar graph (right) shows resorption area quantification (81% reduction; *n* = 5 biologically independent experiments). Scale bars: 25 µm. (C) MA plot of RNA‐seq DEGs in BMDMs after osteoclast induction ± siTREM2@OMVs (fold change ≥1.5, False Discovery Rate (FDR) < 0.05), highlighting downregulated osteoclast genes (Acp5, Ctsk, Nfatc1, Mmp9; red dots). (D,E) GO and KEGG enrichment analysis comparing osteoclasts with or without siTREM2@OMVs treatment. (F) GSEA of the major enriched pathways related to osteoclast differentiation and function. (G) RT‐qPCR of the expression levels of Acp5, Ctsk, Nfatc1, and Atp6v0d2 after treated with siTREM2@OMVs (*n* = 3 biologically independent experiments). (H,I) Schematic (H) of Transwell coculture assay for E0771 invasion through Matrigel toward osteoclasts ± siTREM2@OMVs. Crystal violet staining (I, right) and quantification of invaded cells (59% reduction; *n* = 5 biologically independent experiments). Scale bar: 100 µm. Data were presented as the mean ± SD (unpaired two‐tailed Student's *t*‐test, ****p < 0.0001).

Next, the effects of siTREM2@OMVs on osteoclastogenesis were investigated by using BMDMs to mimic clinical settings. After five days of culture under M‐CSF and RANKL stimulation, osteoclasts are typically characterized by multinucleated and TRAP‐positive cells. TRAP staining showed that significantly less osteoclasts were observed in siTREM2@OMVs groups after osteoclastic differentiation than these in the control group (Figure 5A). Further, bone resorption pit assays were carried out to assess the role of siTREM2@OMVs on the function of osteoclasts. Similarly, siTREM2@OMVs treatment significantly decreased the number and the size of bone resorption pits compared with the control group. The quantitative analysis showed a 81% reduction in siTREM2@OMVs group (Figure 5B). These results indicated that siTREM2@OMVs not only reduced osteoclast formation but also severely impaired their bone resorption activity.

To further investigate the mechanism of siTREM2@OMVs‐mediated suppression of osteoclast differentiation, we performed RNA‐sequencing analysis to compare the transcriptional profiles of BMDMs after 5 days of osteoclast differentiation induced by M‐CSF and RANKL, between the siTREM2@OMVs‐treated group and the untreated control group. The RNA‐sequencing data showed well‐separated gene expression patterns between two groups with a total of 1002 downregulated and 290 upregulated genes in BMDMs in siTREM2@OMVs group, as shown in the heatmap and MA plot (Figure 5C and Figure S23). The DEGs associated with osteoclastogenesis (Acp5, Ctsk, Nfatc1, and Mmp9, etc.) were significantly downregulated in the MA plot (Figure 5C). Gene Ontology (GO) analysis and Kyoto Encyclopedia of Genes and Genomes (KEGG) pathway enrichment analysis revealed that these DEGs in siTREM2@OMVs group were significantly enriched in osteoclast differentiation and bone resorption‐related pathways (Figure 5D,E). Gene set enrichment analysis (GSEA) further revealed that pathways related to osteoclast differentiation were suppressed in siTREM2@OMVs group, including Rap1 signaling pathway, PI3K‐Akt signaling pathway and MAPK signaling pathway (Figure 5F). RT‐qPCR quantitative assessment also demonstrated that the expression of osteoclast‐specific marker genes (Acp5, Ctsk, Nfatc1, and Atp6v0d2) was significantly downregulated by siTREM2@OMVs treatment, confirming that silencing TREM2 significantly inhibits the expression of critical genes driving osteoclast differentiation and function (Figure 5G).

To further verify the effect of inhibiting osteoclast differentiation by TREM2 silencing on tumor invasiveness, we then performed a transwell assay to test the invasion of tumor cells cocultured with osteoclasts and to simulate tumor invasion of the bone surface (Figure 5H). Quantitative analysis revealed that siTREM2@OMVs treatment greatly inhibited the tumor invasion and the number of invasive cells decreased by 59% compared with the control group, demonstrated that the suppression of osteoclast differentiation and function by siTREM2@OMVs translates into a significant reduction in tumor cell invasiveness (Figure 5I).

### 2.6 In Vivo Antitumor Effects of siTREM2@ETP‐PEOz‐OMVs in TNBC Bone Metastasis

**FIGURE 6:**
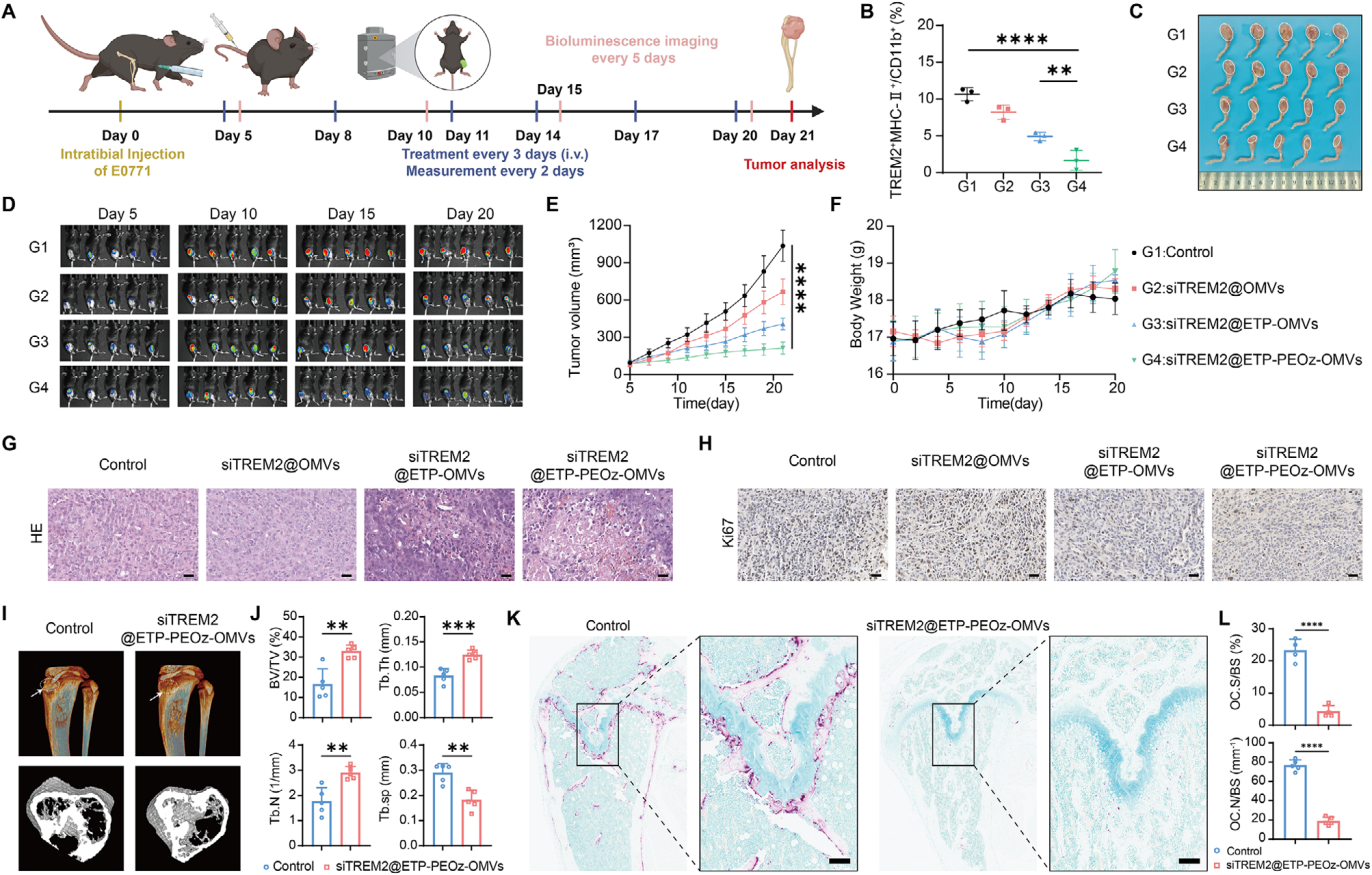
In Vivo Antitumor Capacity of siTREM2@ETP‐PEOz‐OMVs. (A) Schematic illustration of siTREM2@ETP‐PEOz‐OMVs‐mediated antitumor experiment in the TNBC bone metastases modeling. (B) Flow cytometry analysis of TREM2 expression level in TAMs of TNBC female mice with indicated treatment (*n* = 3 biologically independent experiments). Gating strategies as in Figure S6H. (C) The photographs of excised tumors collected on day 21 (*n* = 5 biologically independent experiments). (D) Bioluminescence images of E0771 tumor‐bearing mice at Day5, 10, 15, and 20 posttumor inoculation. (E,F) Tumor volume and body weight changes after i.v. administration of different OMVs (*n* = 5 biologically independent experiments). (G,H) H&E staining (G) of tumor sections showing sparse cells and necrosis in siTREM2@ETP‐PEOz‐OMVs group vs. controls. Ki67 immunohistochemistry (H) shows reduced proliferation (brown nuclei). Scale bars: 20 µm. (I) Micro‐CT 3D reconstructions of tibias from tumor‐bearing mice ± treatments, showing preserved bone structure in siTREM2@ETP‐PEOz‐OMVs group. (J) The statistical analysis of BV/TV, Tb·N, Tb·Th, and Tb.Sp of the proximal tibia from mice treated with siTREM2@ETP‐PEOz‐OMVs as indicated (*n* = 5 biologically independent experiments). (K) TRAP staining of tumor–bone interface and quantification of osteoclast number per bone surface (OC.N/BS) and osteoclast surface area per bone surface (OC.S/BS) (reduced in treated group; *n* = 5 biologically independent experiments). Data were presented as the mean ± SD (unpaired two‐tailed Student's *t*‐test, **p < 0.01, ***p < 0.001, ****p < 0.0001).

To systematically evaluate the therapeutic potential of our hierarchical targeting system, we established a tibial orthotopic TNBC model using E0771‐luciferase cells in female mice. In order to investigate the antitumor effects of different preparations, the mice were randomly divided into 4 groups and the mice were intravenously injected with PBS, siTREM2@OMVs, siTREM2@ETP‐OMVs or siTREM2@ETP‐PEOz‐OMVs once every three days (Figure 6A). We first confirmed through flow cytometry that TREM2 expression in myeloid cells was significantly downregulated in tumors after treatment (Figure 6B), demonstrating the excellent silencing effect of the delivery system in vivo. Tumor volume was also measured and recorded every 2 days after the first treatment. We recorded tumor progression in each group of mice via in vivo imaging. Compared to PBS‐treated mice, the bioluminescence signals of the siTREM2@ETP‐PEOz‐OMVs treated mice were significantly reduced posttreatment (Figure 6D). After the fifth dose of therapy, tumor tissues were harvested after mice were sacrificed. As shown in Figure 6C, mice administered siTREM2@ETP‐PEOz‐OMVs exhibited more effective inhibition of malignant cell proliferation compared to those treated with siTREM2@OMVs, demonstrating the superior in vivo antitumor capacity of siTREM2@ETP‐PEOz‐OMVs. Tumor volume was measured and recorded every two days after the first treatment. The siTREM2@ETP‐PEOz‐OMVs group exhibited the slowest tumor growth trend (Figure 6E), indicating that targeted inhibition of TREM2 expression in monocytes effectively suppresses the growth of TNBC bone metastasis tumors.

The biosafety profile of OMVs was assessed at both the systemic and organ levels. Systemically, mice showed no significant weight loss, and their hematological and blood biochemical profiles remained normal (Figure 6F and Figure S24). At the organ level, hematoxylin and eosin (H&E) staining revealed no histopathological abnormalities. This combination of unaffected physiological parameters and normal tissue morphology confirms the good biocompatibility of the OMVs (Figure S25). H&E staining of tumor tissues revealed that siTREM2@ETP‐PEOz‐OMVs‐treated mice exhibited the most sparsely arranged tumor cells with increased interstitial space compared to other groups, alongside classic cellular necrosis features such as karyolysis and nuclear pyknosis, collectively demonstrating potent antitumor efficacy (Figure 6G). Immunohistochemical staining of Ki67 revealed that treatment with siTREM2@ETP‐PEOz‐OMVs significantly reduced tumor cell proliferative activity in bone metastasis (Figure 6H). Bone structural analysis showed that this treatment also ameliorated structural damage and reduced bone volume loss in the proximal tibial metaphysis (Figure 6I). Furthermore, the siTREM2@ETP‐PEOz‐OMVs group exhibited notably reduced bone volume fraction (bone tissue volume/total tissue volume, BV/TV), trabecular thickness (Tb.Th), and trabecular number (Tb.N), with a marked increase in trabecular separation (Tb.Sp) (Figure 6J), indicating reduced bone erosion associated with bone metastasis. In addition, histological analysis of serial sections revealed abundant TRAP‐positive osteoclasts at the tumor–bone interface, which were substantially decreased after siTREM2@ETP‐PEOz‐OMVs treatment, suggesting effective inhibition of osteoclast formation during bone metastasis progression (Figure 6K,L). These results demonstrate that our platform achieves robust therapeutic efficacy by disrupting the vicious cycle in TNBC bone metastasis while maintaining excellent safety suitable for clinical translation.

### 2.7 siTREM2@ETP‐PEOz‐OMVs Reprogram the Immunosuppressive TME by Regulating the TAM Phenotype and Promoting T‐Cell Infiltration

**FIGURE 7:**
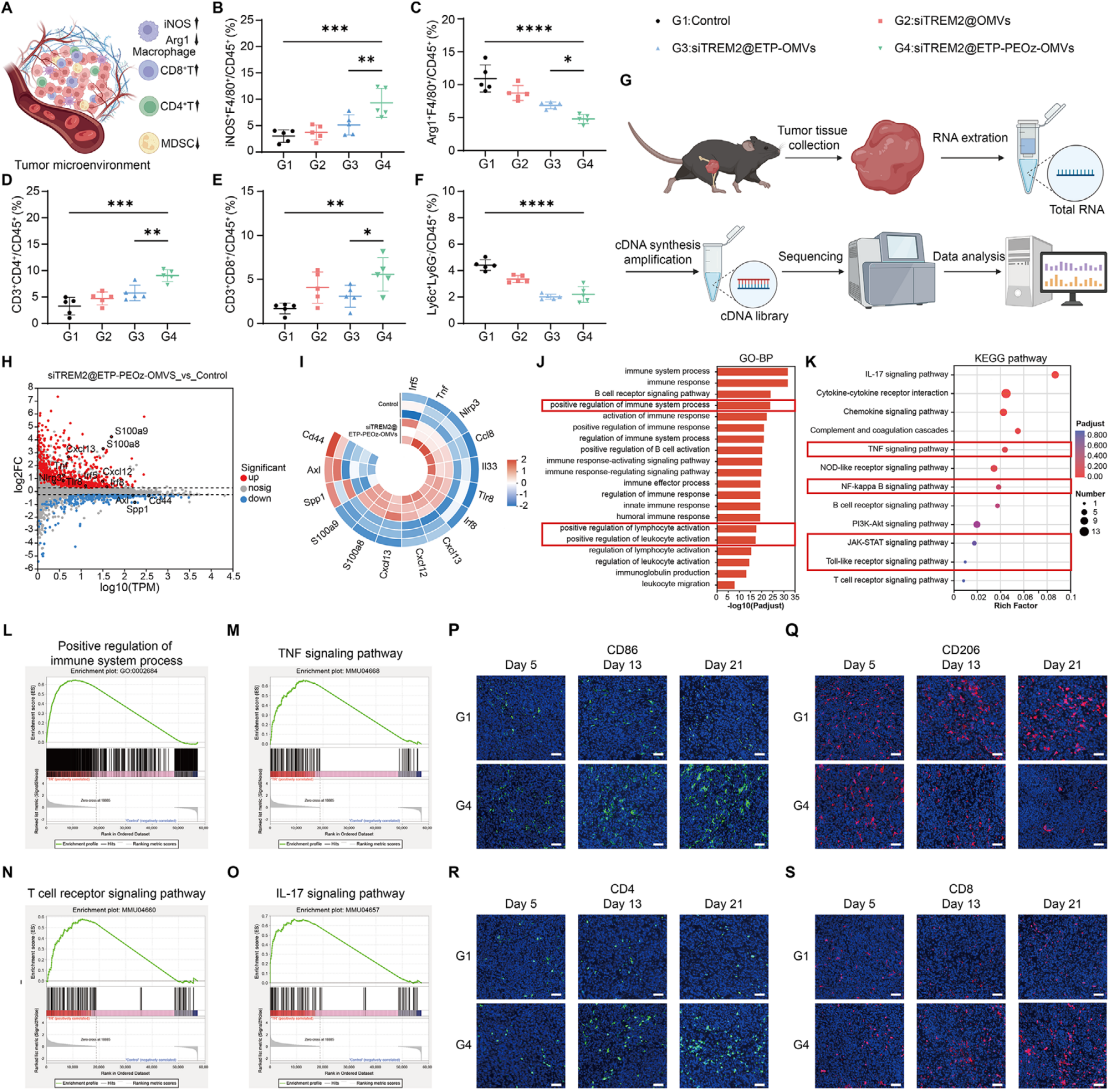
siTREM2@ETP‐PEOz‐OMVs alleviated the immunosuppressive TME by regulating the TAM phenotype and enhancing T cell infiltration. (A) Schematic diagram of the regulatory effect of siTREM2@ETP‐PEOz‐OMVs on the immunosuppressive TME, indicating an increase about immune‐active cells and a decrease in the proportion of immune‐suppressing cells within the TME. (B–F) Flow cytometry quantification of iNOS^+^ F4/80^+^ (B), Arg1^+^ F4/80^+^ (C) macrophages, CD3^+^CD4^+^ (D), CD3^+^CD8^+^ (E) T cells, and Ly6C^+^Ly6G^−^ M‐MDSCs (F) in tumors from treated groups (*n* = 5 biologically independent experiments). Gating strategies as in Figure S6A–C. (G) Schematic workflow of RNA‐seq analysis for tumor tissues from tumor‐bearing mice. (H) MA plot of RNA‐seq DEGs in tumors ± siTREM2@ETP‐PEOz‐OMVs (FDR < 0.05, |log2FC|>1.2). (I) Gene expression circle heatmap across different groups for the DEGs. Heatmap colors indicate normalized gene expression ranging from high (red) to low (blue). (J,K) GO and KEGG enrichment analysis reveals the biological functions or immune‐related pathways that are enriched in the significantly up‐regulated expressed genes. (L–O) GSEA to analyze DEG between the Control group and siTREM2@ETP‐PEOz‐OMVs group. (P–S) CD86, CD206, CD4, and CD8 fluorescent staining of tumors extracted from mice at Day 5, 13, and 21 with various treatments. Scale bars: 200 µm. Data were presented as the mean ± SD (one‐way ANOVA comparisons tests, *p < 0.05, **p < 0.01, ***p < 0.001, ****p < 0.0001).

The proportion of tumor‐infiltrated immune cells (proinflammatory macrophage, immunosuppressive macrophage, CD4^+^ T cell, CD8^+^ T cell, and M‐MDSC) in tumor tissues collected on Day 21 were used to monitor the changes in immune status of the TME (Figure 7A). Recent preclinical and clinical insights indicated that macrophages were the most abundant nonneoplastic critical effector cells of immunotherapy in the microenvironment of TNBC bone metastasis [28, 59]. siTREM2@ETP‐PEOz‐OMVs treatment was shown to induce profound immunomodulatory effects, driving a 3.07‐fold increase in tumoricidal iNOS^+^F4/80^+^ macrophages while reducing immunosuppressive Arg1^+^F4/80^+^ populations by 56% compared to controls (Figure 7B,C), demonstrating its ability to regulate macrophage immunosuppressive function in TNBC bone metastases, in alignment with in vitro findings. Tumor‐infiltrating lymphocytes (TILs), particularly CD4^+^ and CD8^+^ T cell, are key components of the TME in breast cancer, with their increased density associated with improved clinical outcomes in TNBC [60, 61, 62], prompting further evaluation of intratumoral T cell subtype proportions. The proportions of CD3^+^CD4^+^ T cells (Figure 7D) and CD3^+^CD8^+^ T cells (Figure 7E) in the siTREM2@ETP‐PEOz‐OMVs group increased by 179% and 229% respectively compared with the control group, indicating that the proinflammatory polarization of TAMs enhanced T cell infiltration in TNBC bone metastasis tumor tissues. Concurrently, the proportion of M‐MDSCs in the siTREM2@ETP‐PEOz‐OMVs group decreased by 50% (Figure 7F), suggesting that it also participates in enhancing T cell‐mediated adaptive immune responses by reducing myeloid immunosuppression.

Considering the role of PEOz‐mediated sequentially targeting, we focused on comparing the immune cell changes between siTREM2@ETP‐OMVs group and siTREM2@ETP‐PEOz‐OMVs group. Significantly, the siTREM2@ETP‐PEOz‐OMVs group exhibited higher proportions of iNOS^+^F4/80^+^ macrophages, effector CD4^+^ T cells, CD8^+^ T cells, and lower proportions of Arg1^+^F4/80^+^ macrophages than the siTREM2@ETP‐OMVs group, indicating a further enhanced antitumor immune microenvironment in TNBC bone metastatic lesions through PEOz‐mediated sequential targeting (Figure 7B–E).

To elucidate the mechanism by which siTREM2@ETP‐PEOz‐OMVs remodels the immunosuppressive microenvironment, we performed RNA‐sequencing analysis of tibial tumors at Day 21. This revealed profound transcriptomic reprogramming, with over 600 DEGs (Figure 7H). A heatmap of these DEGs confirmed the consistent and pronounced shift in the global transcriptional landscape following treatment (Figure S26). To extract biological meaning from this complex dataset, we first employed GO enrichment analysis and immediately highlighted a dominant theme: the treated tumors were markedly enriched for processes fundamental to immune activation, including ‘positive regulation of immune system process’, ‘leukocyte activation’, and ‘lymphocyte activation’ (Figure 7J). This provided a high‐level, functional confirmation of a profound shift toward immune activation.

We next sought to define the specific signaling architecture driving this response, we performed KEGG pathway analysis, which identified the key modulated pathways (Figure 7K). The response is initiated by the activation of core signaling hubs, including the TNF, NF‐κB, JAK‐STAT, and Toll‐like receptor pathways. These pathways, responsible for initial proinflammatory signal transduction, were reflected in the upregulation of key genes such as Tnf, Nlrp3, Irf5, Irf8, and Tlr8 (Figure 7I). This primary signal then engages broad innate immune effector systems, such as the IL‐17 pathway, evidenced by the upregulation of alarmins like S100a8/a9. Concurrently, and critically, major immunosuppressive axes are dismantled, as seen in the dramatic downregulation of factors like Spp1, Axl, and Cd44. The resultant proinflammatory, desuppressed milieu logically fosters adaptive immune recruitment and coordination. This is supported by the enrichment of the T cell receptor signaling pathway and the significant upregulation of T cell chemokines like Cxcl12 and Cxcl13 (Figure 7I). The overarching importance of these immune coordination networks was further solidified by GSEA, which confirmed the significant enrichment of gene sets for positive regulation of immune system process, TNF signaling pathway, T cell receptor signaling pathway, and IL‐17 signaling pathway (Figure 7L–O). Critically, the expression changes of key genes across this cascade (e.g., Tnf, Stat1, Nfkbia, Tlr8, S100a8, Irf8, Cxcl13, and Spp1) were independently validated via qPCR (Figure S27). Collectively, siTREM2@ETP‐PEOz‐OMVs treatment triggers a coordinated biological program. This program progresses from the activation of core inflammatory signaling hubs, through the engagement of innate effector functions and the reversal of suppression, to the promotion of adaptive immunity, thereby comprehensively reprogramming the TME toward an antitumor state.

To comprehensively analyze the immune cell composition at different stages of tumor progression and further validate the changes in the transcriptomic profiles, we performed immunofluorescence staining analysis on tibial tumor tissues at days 5, 13, and 21 posttreatments. As shown in Figure 7P,Q, staining results revealed a progressive increase in CD86 positive macrophages and the corresponding decrease in CD206 positive macrophages in the siTREM2@ETP‐PEOz‐OMVs‐treated group. Likewise, effector CD4^+^ and CD8^+^ T cell infiltration in tibial tumor tissues of the treated group notably increased (Figure 7R,S). The specificity of all immunofluorescence staining was confirmed by negative controls, which showed no detectable signal (Figure S28). Collectively, these findings indicate that TREM2 silencing enhances M1‐like macrophage polarization and effector T cell infiltration, strongly promoting antitumor immunity. siTREM2@ETP‐PEOz‐OMVs treatment disrupts the TGF‐β/IL‐10‐mediated immunosuppressive cycle between TREM2^+^ TAMs and tumor cells, fostering an antitumor immune microenvironment in TNBC bone metastases and inhibiting tumor progression by reducing osteoclast activity.

## 3 Discussion

Our findings establish that in the TNBC bone metastatic niche, TREM2 functions as a master regulator orchestrating the differentiation of monocyte precursors into both immunosuppressive TAMs and osteoclasts [20, 21, 22]. Silencing TREM2 thus delivers a dual therapeutic impact: it repolarizes macrophages toward a proinflammatory phenotype and suppresses osteoclast‐mediated bone resorption. This simultaneous action directly disrupts the core immunosuppressive‐osteolytic cycle, fostering an antitumor immune microenvironment.

The realization of this myeloid targeting strategy required a delivery platform capable of navigating the biological hierarchy of the metastatic niche. Our siTREM2@ETP‐PEOz‐OMVs system meets this challenge through a purposefully integrated design that advances beyond existing nanotechnologies in several key aspects. While siRNA delivery to myeloid cells has been attempted using ligand‐decorated nanoparticles, and while pH‐responsive nanomaterials have been widely explored, prior systems often address only one barrier in isolation [39, 63, 64, 65]. Our platform pioneers tailored design strategies for complex biological interactions involved in TNBC bone metastasis. The initial tumor accumulation is mediated by the phage display‐derived ETP peptide, ensuring high local concentration [47]. Upon encountering the acidic TME, the pH‐sensitive PEOz coating dissociates, unveiling the intact OMV carrier. This critical transition is not a simple drug release, but the activation of a stealth carrier into a targetable one, leveraging the OMV's innate tropism for myeloid cells via TLR4 interactions—a targeting mechanism that is more efficient and biologically ingrained than many engineered ligands. Consequently, the potent siTREM2 is selectively delivered to myeloid populations within the tumor. This hierarchical targeting cascade—from tissue to cell—ensures that the therapeutic payload is activated precisely where and when needed, minimizing off‐target effects and systemic exposure by confining the immunomodulatory and antiosteolytic actions synergistically to the disease site.

Our strategy diverges significantly from prior approaches targeting TREM2 or myeloid reprogramming. Existing therapeutic efforts have largely focused on modulating the polarization state of terminally differentiated macrophages (e.g., driving M2‐to‐M1 repolarization) as a means to alleviate immunosuppression [66, 67, 68]. While effective in some contexts, such “end‐point” interventions often overlook the plasticity and shared ontogeny of myeloid lineages within the metastatic niche [7, 69]. By silencing TREM2 within bone‐infiltrating myeloid populations, we instead achieve a fate‐constrained reprogramming that simultaneously alleviates immunosuppression and suppresses osteoclastogenesis. Functionally, this upstream intervention resulted in a 2.7‐fold increase in iNOS^+^F4/80^+^ proinflammatory macrophages, a 58% reduction in Arg1^+^F4/80^+^ immunosuppressive macrophages, a 123% expansion of cytotoxic CD8^+^ T cells, a 46% increase in CD4^+^ T cells, and a 39% decrease in monocytic MDSCs within bone metastatic lesions. In parallel, osteoclast formation at the tumor–bone interface was markedly reduced, with >80% inhibition of osteoclast differentiation and bone resorption activity in vitro. These coordinated immune‐restorative and antiosteolytic effects translated into a 79.5% suppression of osteolysis‐driven tumor progression and significantly reduced tumor cell proliferation in bone lesions. Collectively, these data demonstrate that intervening upstream at the TREM2‐controlled differentiation fork enables simultaneous remodeling of the immune microenvironment and osteolytic niche, thereby dismantling the self‐reinforcing vicious triangle among tumor cells, immunosuppressive TAMs, and osteoclasts—an outcome unattainable by strategies targeting macrophage polarization or bone resorption alone.

While our findings highlight the promising therapeutic potential of siTREM2@ETP‐PEOz‐OMVs, several translational considerations warrant discussion. Firstly, compared to current standard clinical therapies for TNBC bone metastases—primarily consisting of systemic chemotherapy, bone‐modifying agents (e.g., bisphosphonates or denosumab), and limited immunotherapy options [70, 71]—our platform offers a dual mechanism simultaneously targeting the immunosuppressive niche and osteolytic progression, a strategy not addressed by conventional monotherapies. However, its clinical efficacy and safety relative to these established treatments remain to be validated in comparative preclinical models and, ultimately, clinical trials. Regarding potential toxicity, although OMVs derived from engineered bacteria can be designed with reduced virulence and modified lipid composition to minimize endotoxin‐related effects, their inherent immunogenicity may elicit inflammatory responses or preexisting immunity in some patients [72]. This could potentially affect delivery efficiency or induce adverse effects, underscoring the need for rigorous toxicity profiling and possibly the use of autologous macrophage‐derived vesicles to mitigate immune recognition [73]. Furthermore, while the ETP peptide aims to enhance tumor targeting, its specificity across heterogeneous TNBC subtypes requires further optimization to avoid off‐target accumulation, particularly in critical organs [74]. These limitations highlight the importance of integrating comprehensive biodistribution, dose‐ranging toxicology studies, and immunogenicity assessments in future development stages.

The dual suppression of immunosuppressive TAMs and osteoclasts by siTREM2@ETP‐PEOz‐OMVs offers a translatable paradigm for TNBC bone metastases, where therapies remain limited. The platform's components, including ETP peptides and OMVs, can be industrially produced and stably stored, requiring only simple incubation to administration, thus bridging standardized and personalized treatment approaches. This strategy reduces patient waiting times and supports the potential commercialization of TREM2‐targeted therapies. Future investigations should explore combining siTREM2@ETP‐PEOz‐OMVs with immune checkpoint inhibitors to further amplify T cell responses and validate efficacy in patient‐derived xenograft models to enhance clinical translation.

## 4 Materials and Methods

### 4.1 Single‐Cell RNA Sequencing Data Processing and Analysis

Fresh bone metastatic tumor tissues were surgically resected from four patients with pathologically confirmed TNBC at our institution for single‐cell RNA sequencing analysis. Single‐cell RNA sequencing was performed on cells isolated from bone metastasis lesions of 4 patients with TNBC. We first filtered the matrices to exclude low‐quality cells using a standard panel of three quality criteria: (1) number of detected transcripts (number of unique molecular identifiers); (2) detected genes; and (3) percent of reads mapping to mitochondrial genes (The numbers out of the limit of mean value ± 2‐fold of standard deviations). The expression of mitochondria genes was calculated using PercentageFeatureSet function of the Seurat package. The normalized data (NormalizeData function in Seurat package) was performed for extracting a subset of variable genes. Variable genes were identified while controlling for the strong relationship between variability and average expression. For batch correction across the 4 patient samples and subsequent graph‐based clustering, the processed data were imported into Scanpy (v1.8.2). Batch effects were corrected using sc.pp.combat with Harmony algorithm (dims = 1:30). Principal component analysis (PCA) was performed, and a neighborhood graph was built using the top 30 principal components. Clustering was conducted using the Leiden algorithm with a resolution of 0.6, which was selected after testing a range of parameters and was found to optimally separate major cell types and yield biologically interpretable myeloid subpopulations. Cell types were annotated based on canonical marker genes. Trajectory analysis for the monocyte‐to‐macrophage/osteoclast lineage was performed on subsetted myeloid cells using Monocle2 (v2.18.0), with pseudotime inferred from the top 2000 variable genes and DEGs identified using Wilcoxon rank‐sum test (adjusted *p* < 0.05, log2 fold change > 0.25).

### 4.2 Reagents

The antibodies used for immunofluorescence imaging were the following: anti‐mouse CD86 (CST, CAT: **#**19589), anti‐mouse CD206 (CST, CAT: **#**24595), anti‐mouse CD4 (Abcam, CAT: #ab183686), anti‐mouse CD8 (Abcam, CAT: #ab209775), anti‐mouse iNOS (Abcam, CAT: #ab283655), anti‐mouse Arg1 (Proteintech, CAT: #16001‐1‐AP). The antibodies used for flow cytometry were the following: Zombie UV Fixable Viability Kit (BioLegend, USA, Cat: #423107), anti‐mouse CD45 BV421 (BioLegend, Cat: #147719), anti‐mouse CD45 AF700 (BioLegend, Cat: #103127), anti‐mouse CD11b PerCP (BioLegend, Cat: # 101229), anti‐mouse F4/80 APC‐Cy7 (BioLegend, Cat: # 123117), PE/Cyanine7 anti‐Nos2 (iNOS) Antibody (BioLegend, Cat: # 696813), Arg1 antibody APC (ThermoFisher, Cat: # 17‐3697‐80), anti‐mouse Ly6C APC (BioLegend, Cat: # 128015), anti‐mouse Ly6G FITC (BioLegend, Cat: # 127605), anti‐mouse CD3 BV421 (BioLegend, Cat: # 100227), anti‐mouse CD4 PE (BioLegend, Cat: # 100407), anti‐mouse CD8a Alexa Fluor 594 (BioLegend, Cat: # 100758), anti‐mouse MHC‐II eFlour450 (eBioscience, Cat: # 48‐5321‐82), anti‐mouse TREM2 FITC (eBioscience, Cat: # MA5‐28223), anti‐human CD11b PerCP/Cyanine5.5 (BioLegend, Cat: # 340011), anti‐human CD284 (TLR4) Brilliant Violet 421 (BioLegend, Cat: # 312811), anti‐human CD68 PE (BioLegend, Cat: # 333807), anti‐human CD86 APC (BioLegend, Cat: # 374207), anti‐human CD206 (MMR) APC/Cyanine7 (BioLegend, Cat: # 321119), Human TruStain FcXTM (Fc Receptor BlockingSolution) (BioLegend, Cat: # 422301). The antibodies used for immunohistochemistry were the following: anti‐Ki67(CST, CAT: #9129). Organic solvents, including chloroform and methanol (of analytical grade), were procured from Shanghai Lingfeng Chemical Reagent Co., Ltd. The siRNA specifically targeting TREM2 (denoted as siTREM2, the sense strand sequence is 5ʹ‐GAGGGUGUCAUGUACUUAU (dT)(dT)‐3ʹ, and the antisense strand sequence is 5ʹ‐AUAAGUACAUGACACCCUC (dT)(dT)‐3ʹ) was synthesized by Shanghai Jiman Technology Co., Ltd. DAPI (Cat: # C1006), Did (Cat: # C1039) and CCK‐8 (Cat: # C0037) were purchased from Beyotime (Shanghai, China). DSPE‐PEOz2000 was purchased from Xi'an Ruixi Biological Technology (China). All chemicals were from Zhejiang University's reagent platform.

### 4.3 Mice and Cell Lines

C57BL/6J‐Trem2^em2Adiuj^/J (Trem2^−/−^) mice were purchased from The Jackson Laboratory. Wild‐type male C57BL/6 mice aged 6–8 weeks were provided by the Experimental Animal Center of Zhejiang Province. The mice were housed in an environment with a temperature of 20–22°C and a humidity of 30–70%, with ample access to feed and water. Artificial lighting was provided on a 12 h light/12 h dark cycle. All procedures involving animals were conducted with the approval and under the guidance of the Ethics Committee for Experimental Animals of the Second Affiliated Hospital, Zhejiang University School of Medicine.

The murine E0771 (RRID: CVCL_GR23), RAW264.7 (RRID:CVCL_0493) and 3T3‐L1 (RRID:CVCL_0123) cell lines were obtained from the Cell Collection of the Chinese Academy of Science (Shanghai, China). All cell lines were confirmed mycoplasma negative by the standard polymerase chain reaction method. E0771 cells, RAW264.7 and 3T3‐L1 cells were cultured in Dulbecco's modified Eagle's medium (DMEM; Gibco) with 10% FBS and 1% penicillin/streptomycin (Sigma‐Aldrich). The medium was changed every two days, and all cells were cultured at 37°C in a 5% CO2 atmosphere.

BMDMs were isolated from the bone marrow of 6 weeks old female C57BL/6 mice according to previously published protocols [75]. After isolation, cells were subjected to erythrocyte lysis (Solarbio) to remove the red blood cells and then cultured for 7 days in RPMI 1640 medium supplemented with 10% FBS, 1% penicillin/streptomycin, and 50 ng/mL M‐CSF (Novoprotein).

### 4.4 Osteoclast Differentiation

BMDMs were harvested from the long bones of 6‐week‐old C57BL/6 mice. The cells were cultured in α‐MEM supplemented with 10% FBS and M‐CSF (50 ng/mL) for 2 days. For differentiation, BMDMs were seeded at a density of 2 × 10^5^ cells per well in 12‐well plates and cultured in α‐MEM supplemented with 10% FBS and M‐CSF (50 ng/mL) for 2 days. Subsequently, cells were induced with RANKL (50 ng/mL) for 5 days, with media changed every 2 days (twice daily from Day 5 onward to minimize variability). Following differentiation, cells were fixed with 4% paraformaldehyde (PFA) and stained using a TRAP staining kit (42010102, Sigma‐Aldrich, Shanghai, China). TRAP‐positive cells containing five or more nuclei were identified as mature osteoclasts and quantified using ImageJ software (v1.53), with at least 5 random fields per well analyzed blindly to reduce observer bias (*n* = 5 biologically independent experiments per group).

### 4.5 In Vivo Phage Display

A linear 12‐amino acid peptide library (Ph.D.‐12 phage display peptide library, New England Biolabs, Inc.) was used for biopanning following our previous protocol [76]. First, mouse primary fibroblasts were cocultured with 1 × 10^12^ transducing units of the phage library for negative selection. The supernatant was then collected and incubated with E0771 cells for positive selection. After 1 h, E0771 cells were washed 5 times with a washing buffer to remove unbound phages. The cell‐bound phages were subsequently eluted using an elution buffer. These selected phages were amplified by infecting *E. coli* host strain ER2738 for the next round of selection. Following negative selection, the remaining phages were intravenously injected into mice intratibially injected with 1 × 10^6^ E0771 cells for 3 weeks. One hour later, the mice were sacrificed, and heart perfusion was performed to wash away residual phages from the bloodstream. The tumor tissue was then excised, and tumor‐targeted phages were extracted from the tumor homogenate before being amplified through infection of *E. coli* host strain ER2738.

### 4.6 Synthesis and Dye Labeling of Peptides

The peptides containing four additional lysines (KKKK) at the C‐terminal as a linker region and the palmitoylated peptides were synthesized by KS‐V Peptide Biological Technology Co. Ltd (Hefei, China). For palmitoylated peptides, the Fmoc/tBu solid‐phase peptide synthesis strategy was employed, with palmitic acid specifically conjugated to the ε‐NH2 group of the first C‐terminal lysine residue. Cy5.5 dye conjugation was performed according to our previous methodology [75]: a mixture of Cy5.5‐maleimide (200 µL, 1 mm) and peptides (800 µL, 1 mm) was reacted at room temperature for 3 h under constant stirring. Free unconjugated peptides were subsequently removed using Slide‐A‐Lyzer Dialysis Cassettes (1000 MWCO, Thermo Fisher Scientific, Waltham, MA, USA).

### 4.7 In Vivo Fluorescence Imaging of Selected Peptides

Tumor‐bearing mice were established by injecting 1.0 × 10^7^ E0771 cells in 10 µL of PBS into the left tibial bone marrow cavity. 10 days posttumor inoculation, 100 µL of Cy5.5‐Peptide A, Cy5.5‐Peptide B, and Cy5.5‐Peptide C (50 × 10^−6^ m) were administered via tail vein injection. 24 h postinjection, anesthetized mice (maintained with 5% isoflurane/oxygen) were imaged using an in vivo imaging system (PerkinElmer) with 678 nm excitation and 695 nm emission wavelengths.

### 4.8 Fluorescence Staining of Selected Peptides in Vitro

E0771 cells were cultured on confocal dishes at a density of 2.0 × 10^5^ cells per dish. After blocking with 10% bovine serum albumin for 60 min at 37°C, the cells were incubated with Cy5.5‐labeled Peptide A, Cy5.5‐labeled Peptide B, and Cy5.5‐labeled Peptide C (10 × 10^−6^ m) for 30 min at 37°C under dark conditions. Following three rinses with PBS, the cells were fixed with PFA for 15 min. Subsequently, fixed cells were stained with FITC‐phalloidin (Thermo Fisher Scientific Co., USA) for 2 h and DAPI (Beyotime, China) for 5 min at 37°C. Fluorescence imaging was performed using a confocal microscope with 633 nm excitation and a 700/50 nm emission filter for detection.

### 4.9 RNA Extraction and Real‐Time PCR

Total RNA was isolated using the RNAeasy Animal RNA Isolation Kit with Spin Column (Beyotime, Cat: # R0024). Reverse transcription was performed using a cDNA Synthesis Kit (TaKaRa). Real‐time quantitative PCR was then conducted with SYBR Premix Ex Taq kits (TaKaRa) on an Applied Biosystems 7500 system. Gene expression levels were normalized to β‐actin. All primer sequences were synthesized by Sangon Biotech (Shanghai, China), as listed in Table 1.

**TABLE 1:** Primers used in quantitative RT‐PCR.

Gene	Forward primer (5ʹ to 3ʹ)	Reverse primer (5ʹ to 3ʹ)
β‐actin	CATTGCTGACAGGATGCAGAAGG	TGCTGGAAGGTGGACAGTGAGG
IL‐6	TACCACTTCACAAGTCGGAGGC	CTGCAAGTGCATCATCGTTGTTC
MIP2	CATCCAGAGCTTGAGTGTGACG	GGCTTCAGGGTCAAGGCAAACT
Arg1	CATTGGCTTGCGAGACGTAGAC	GCTGAAGGTCTCTTCCATCACC
IL‐10	CGGGAAGACAATAACTGCACCC	CGGTTAGCAGTATGTTGTCCAGC
TGF‐β	TGATACGCCTGAGTGGCTGTCT	CACAAGAGCAGTGAGCGCTGAA
TREM2	CTACCAGTGTCAGAGTCTCCGA	CCTCGAAACTCGATGACTCCTC

### 4.10 Immunofluorescence

Cells were plated in confocal dishes, washed with PBS, and fixed with 4% PFA at room temperature for 15 min. After fixation, cells were blocked and permeabilized in PBS containing 5% bovine serum albumin (BSA) and 5% rabbit serum for 1 h at room temperature. Primary antibodies diluted in 5% BSA‐PBS were then applied and incubated at 4°C for 1 h. Following three PBS washes, cells were incubated with the corresponding secondary antibodies for 2 h at room temperature in the dark. Nuclei were counterstained with DAPI (1:5000 dilution) for 15 min, followed by three additional PBS washes. Finally, samples were mounted using ProLong Diamond antifade reagent and imaged on a Leica confocal microscope.

### 4.11 Tumor Implantation

For the intratibial injection model, a total of 1 × 10^6^ E0771‐luci cells resuspended in 10 µL PBS were injected intratibially into the left tibial bone marrow cavity of anesthetized mice. After approximately 21 days, tumors were excised and weighed. The tumor volume was calculated as *a* × *b* ^2^ / 2 (a is the largest and b is the smallest diameter).

### 4.12 Bioluminescence Imaging

In vivo bioluminescence imaging was conducted utilizing an in vivo imaging system (AniView system). Mice were anesthetized and then immediately administered an intraperitoneal injection of 100 µL D‐luciferin (15 mg/mL; Invitrogen, CAT: #L2916) prior to image acquisition. Metastatic burden was quantified by calculating total flux within standardized regions of interest through AniView software.

### 4.13 Flow Cytometry

Tumor tissues were enzymatically digested in DMEM supplemented with type IV collagenase (2 mg/mL), deoxyribonuclease (0.1 mg/mL), and hyaluronidase (0.1 mg/mL) (all from Sigma‐Aldrich) at 37°C for 1 h. The digested suspensions were filtered through 70 µm cell strainers to obtain single‐cell suspensions. After washing with PBS, cells were pretreated with TruStain FcX (anti‐mouse CD16/32; BioLegend, CAT: #101319) to block Fc receptors, then costained with Zombie Aqua Fixable Viability Kit and surface marker antibodies to exclude dead cells. For intracellular targets, cells were fixed and permeabilized using the True‐Nuclear Transcription Factor Buffer Set (BioLegend, CAT: #424401) according to manufacturer guidelines, followed by 20 min incubation with specific antibodies in the dark at room temperature. Flow cytometry analysis was conducted on a Beckman Coulter CytoFLEX LX platform with CytExpert v2.4 software, and data were processed using FlowJo v10.

### 4.14 Extraction and Purification of OMVs

*E. coli* (ΔmsbB) was constructed through Inovogen Tech. Co. (Chongqing, China). Bacterial cultures were initiated by inoculating LB medium with 1% v/v of the strain and shaking‐incubated until reaching logarithmic phase (OD600 = 1). Cells were pelleted by centrifugation at 4000× *g* for 10 min, and supernatants were sterilized through 0.45 µm filters (Millipore brand) to remove residual bacteria. OMVs were sequentially enriched by ultracentrifugation (Beckman) at 150 000×g for 2 h at 4°C. Purified OMVs were resuspended in PBS (pH 7.4) for downstream applications. Polymyxin B (PMB, a LPS‐neutralizing antibiotic) was added to the OMV suspension to a final concentration of 100 µg/mL and incubated at room temperature for 2 h with gentle mixing [77]. The PMB‐treated OMV suspension was ultracentrifuged again at 150 000×g for 2 h at 4°C to remove unbound PMB and impurities. The pelleted material was resuspended in PBS to prepare the OMVs stock solution (samples designated for zeta potential analysis were resuspended in double‐distilled water). The OMVs concentration, defined by the protein concentration of the preparation, was finally quantified using a bicinchoninic acid (BCA) protein assay kit.

### 4.15 Preparation of siTREM2‐Loading OMVs (siTREM2@OMVs)

siTREM2 was loaded into OMVs via electroporation using the Gene Pulser Xcell system (Bio‐Rad, USA). A mixture containing approximately 1 × 10^9^ nanovesicles (quantified by Flow NanoAnalyzer, NamoFCM N30E) and 1 µg siRNA in 400 µL electroporation buffer was transferred to a 4 mm cuvette. Electroporation was performed at 250 V with 250 µF capacitance and a single pulse to generate siTREM2‐loaded OMVs. Residual free siTREM2 was subsequently removed by Sephadex G‐50 gel chromatography (flow rate 0.5 mL/min) under sterile conditions to prevent contamination. Postloading, OMVs were characterized by NTA (ZetaView, Particle Metrix) to confirm size distribution (<200 nm) and purity (>95%), with endotoxin levels verified using Limulus Amebocyte Lysate assay (below 0.1 EU/mL to ensure biosafety).

### 4.16 siTREM2 Encapsulation Efficiency and Loading efficiency

The EE and LE of siTREM2 was assessed using Quant‐iT RiboGreen RNA Assay (Invitrogen). Briefly, siTREM2@OMVs were resuspended in Tris‐EDTA (TE) buffer. To measure the concentration of free siRNA, an aliquot of this suspension was mixed with the RiboGreen reagent directly. To measure the total siRNA, an equal aliquot was first treated with Triton X‐100 at a final concentration of 1% v/v) to completely disrupt the OMV membranes, releasing all siRNA, prior to the addition of the dye.

Standard curves were generated using known concentrations of free siRNA in both plain TE buffer and TE buffer containing 1% Triton X‐100, to account for any potential effects of the detergent on fluorescence. After a 5 min incubation in the dark at room temperature, fluorescence was measured (excitation/emission = 485/535 nm). The siRNA concentrations in the experimental samples were interpolated from their respective standard curves. The masses of siRNA (*W*_total_ and *W*_free_) were calculated by multiplying the concentrations interpolated from the standard curves by the corresponding sample volumes. EE and LE were then calculated as follows:

$$\;\mathrm{EE}\;(\%)\;=\frac{W_{\mathrm{total}}-W_{\mathrm{free}}}{W_{\mathrm{total}}}\;\times 100\%$$

$$\;\mathrm{LE}\;(\%)\;=\frac{W_{\mathrm{total}}-W_{\mathrm{free}}}{W_{\mathrm{OMVs}}}\;\times 100\%$$

where *W*_total_ and *W*_free_ are the masses of total and free siRNA, respectively, and *W*_OMVs_ is the total protein mass of the OMVs used for loading, as determined by the BCA assay.

### 4.17 Preparation and Characterization of siTREM2@ETP‐PEOz‐OMVs

We utilized DSPE‐PEOz2k‐ETP as the nanovesicle membrane anchoring platform, purchased from Xi'an Qiyue Biological Technology Co. The DSPE‐PEOz2k‐ETP was synthesized as follows: 100 mg DSPE‐PEOz‐NHS was dissolved in 3 mL dimethylformamide (DMF), followed by addition of peptide at 1.1 molar equivalents and triethylamine at 3.0 molar equivalents. The reaction proceeded for 12 h at room temperature. The mixture was transferred into a dialysis bag (2 kDa molecular weight cutoff) and dialyzed against purified water for 24 h. The final product was obtained by collecting the dialysate and lyophilization. The resulting complex was then incubated with siTREM2@OMVs (approximately 2 × 10^7^ particles per milliliter, 500 µL) for 20 min at 37°C to embed in the membrane of OMVs. The resulting suspension can be concentrated using a 100 kDa ultrafiltration centrifuge tube.

The sample morphology of OMVs, siTREM2@OMVs, and siTREM2@ETP‐PEOz‐OMVs was observed by the TEM by (JEM‐1200EX transmission electron microscope, Japan). NTA was also used to measure the size distribution of OMVs, siTREM2@OMVs, and siTREM2@ETP‐PEOz‐OMVs (Particle Metrix, zetaview). Zeta potential of nanovesicles were measured using dynamic light scattering (DLS) (Mastersizer 3000, UK). The UV–vis absorbance of different nanovesicles was measured by SHIMADZU UV‐3600 Plus (Japan).

### 4.18 Fluorescence Staining of OMVs In Vitro

To validate the stronger phagocytosis of OMVs by macrophages compared to extracellular vesicles of other origins, RAW 264.7 cells were cultured on confocal dishes (2.0 × 10^5^ cells), and after the cells were sufficiently adhered to the wall, the cells were incubated with Did labelled‐OMVs, Did labelled‐K7M2 EVs and Did labelled‐ 4T1 EVs (100 µL, 1 µg/mL), respectively, after the cells were fully adhered, the cells were incubated with Did labelled‐OMVs, Did labelled‐K7M2 EVs, and Did labelled‐4T1 EVs (100 µL, 1 µg/mL) for 30 min at 37°C in the dark. After three washes with PBS, cells were fixed with PFA for 15 min. Then the fixed cells were incubated with FITC‐phalloidin (Thermo Fisher Scientific Co., USA) for 2 h and DAPI (Beyotime, China) for 5 min at 37°C and observed under a confocal fluorescence microscope (sensors were excited at 644 nm and the red emission was collected using a 663 nm filter set).

To verify stronger specific phagocytosis of OMVs by monocytes/macrophages, RAW 264.7 cells, E0771 cells and 3T3L1 cells were cultured separately on confocal dishes (2.0 × 10^5^ cells). 100 µL of Did‐labelled OMVs (1 µg/mL) was added and the cells were incubated for 1 h at 37°C in the dark. After rinsed for 3 times with PBS, cells were fixed with PFA for 15 min. Then the fixed cells were incubated with FITC‐phalloidin (Thermo Fisher Scientific Co., USA) for 2 h and DAPI (Beyotime, China) for 5 min at 37°C and observed under a confocal fluorescence microscope (sensors were excited at 644 nm and the red emission was collected using a 663 nm filter set).

### 4.19 FTIR Spectroscopy Analysis

DSPE‐PEOz‐ETP samples (5 mg) were dissolved in 1 mL PBS at pH 7.4 or pH 5.4 and incubated at 37°C for 30 min to simulate physiological and acidic TME conditions, respectively. Following incubation, the samples were freeze‐dried to obtain dry powders. FTIR spectra were recorded using a Nicolet iS50 FTIR spectrometer (Thermo Fisher Scientific) in the wavenumber range of 4000–400 cm^−^^1^, with a spectral resolution of 4 cm^−^^1^ and an average of 32 scans per sample. Samples were prepared as KBr pellets by homogeneously mixing 1 mg of the lyophilized powder with 100 mg of spectroscopic‐grade KBr and pressing into transparent discs. Spectral data were analyzed for characteristic changes, such as the diminution of the amide C═O stretch peak at ∼1640 cm^−^^1^ and the emergence of a broad O─H stretch at ∼3400 cm^−^^1^, indicative of acid‐triggered hydrolysis.

### 4.20 siRNA Stability

To assess the extent of protection provided by the nanovesicles to siRNA, the stability of siTREM2 loaded within the nanovesicles against serum degradation was evaluated by gel electrophoresis. Free siTREM2 and siTREM2@ETP‐PEOz‐OMVs were incubated with 10% FBS at 37°C, and samples were collected at different time intervals (0, 2, 4, 6, 8, 10, and 12 h). To dissociate the siTREM2 from the nanovesicles, Triton X‐100 was added. RNA gel electrophoresis typically involves loading the mixture into agarose gel wells, separation by electrophoresis, visualization through staining, and assessment of RNA quality by observing band patterns.

### 4.21 Cell Cytotoxicity Evaluation

The cytotoxicity of nanoparticles was evaluated in vitro using the CCK‐8 assay. RAW 264.7 cells were plated in 96‐well plates at 5 × 10^3^ cells per well and allowed to adhere overnight. Cells were then exposed to increasing concentrations (0.1–2 µg/mL) of nanovesicles (OMVs or siTREM2@ETP‐PEOz‐OMVs) for 24 h. Cell viability was determined by CCK‐8 assay following the manufacturer's protocol.

### 4.22 In Vivo Fluorescence Imaging of siTREM2@ETP‐PEOz‐OMVs

To establish the orthotopic tumor model, 1 × 10^6^ E0771‐luci cells suspended in 10 µL PBS were injected into the left tibial bone marrow cavity of anesthetized mice. Ten days postimplantation, tumor‐bearing mice received 100 µL Did‐labeled siTREM2@OMVs or siTREM2@ETP‐PEOz‐OMVs (100 µg dose) via tail vein injection. Whole‐body fluorescence imaging was performed at scheduled timepoints (3, 6, 9, 12 h postinjection) using an in vivo Imaging System under 5% isoflurane/oxygen anesthesia. At 24 and 72 h, after anesthesia, mice were perfused with 4% PFA, and major organs of mice were excised for ex vivo imaging.

### 4.23 In Vivo Pharmacokinetic Assay

C57BL/6J mice bearing orthotopic TNBC bone metastases received injections of siTREM2@OMVs (5 mg/kg) as well as siTREM2@ETP‐PEOz‐OMVs (5 mg/kg). Blood samples were collected at different intervals of 0.5, 3, 6, 12, and 24 h after the injections and an in vivo imaging system (PerkinElmer) was utilized to monitor the fluorescence intensity. Pharmacokinetic curves were fitted using GraphPad Prism, and relevant parameters were calculated.

### 4.24 Fluorescence Staining of siTREM2@ETP‐PEOz‐OMVs In Vitro

E0771 cells were seeded on confocal dishes at 2.0 × 10^5^ cells per dish for 1–2 days to reach 50–70% confluency. Cells were incubated with 2 mL PBS containing Did‐labeled siTREM2@OMVs (1 µg/mL) or siTREM2@ETP‐PEOz‐OMVs (1 µg/mL) in an atmosphere of 5% CO2 and 95% air for 30 min at 37°C. After three washing steps, fixed cells fixed cells were incubated with DAPI for 5 min at 37°C and observed under a confocal fluorescence microscope (Leica TCS SPSII).

### 4.25 pH‐Sensitive Hierarchical Targeting of siTREM2@ETP‐PEOz‐OMVs

To assess macrophage uptake of siTREM2@ETP‐PEOz‐OMVs at different pH, E0771 cells and Raw 264.7 cells were coinoculated in six‐well plates at a density of 5 × 10^4^ /well each, incubated at 37°C for 24 h and observed under a microscope until 80–90% fusion was achieved. After three washing steps, the cells were incubated with 2 mL of DMEM containing Did‐labelled siTREM2@ETP‐PEOz‐OMVs (1 µg/mL) in 2 mL of DMEM with 10%FBS at 37°C for 1 h. Cells from the different treatment groups were then harvested, washed, and resuspended in PBS to analysis. Flow cytometry was used to quantify the phagocytic uptake of OMV by macrophages and the associated fluorescence intensity.

To validate the complete hierarchical targeting process of siTREM2@ETP‐PEOz‐OMVs by macrophages at different pH, both RAW 264.7 cells and CFSE‐stained E0771 cells were cocultured in confocal dishes at 1.0 × 10^5^ per dish for 1–2 days to achieve 50% fusion. After three washing steps, the cells were incubated with 2 mL of DMEM with 10% FBS (pH 7.4) containing Did‐labelled siTREM2@ETP‐PEOz‐OMVs (1 µg/mL) at 37°C and firstly observed under a confocal fluorescence microscope after 15 min. The pH was then adjusted to 5.4 with 0.1 mm hydrochloric acid solution and observed under confocal fluorescence microscope at different time points (1 and 3 h).

### 4.26 Flow Cytometry Analysis of OMV Uptake in Tumor Tissues

Tibial tumor tissues were collected from E0771 bone metastasis–bearing mice 24 h after intravenous administration of Dio‐labeled siTREM2@ETP‐PEOz‐OMVs (5 mg/kg). The tumor tissues were minced into small fragments and enzymatically digested in DMEM containing type IV collagenase (2 mg/mL), deoxyribonuclease (0.1 mg/mL), and hyaluronidase (0.1 mg/mL) at 37°C for 1 h with gentle agitation. The resulting cell suspensions were filtered through a 70 µm cell strainer and washed with PBS to obtain single‐cell suspensions.

Cells were then incubated with fluorophore‐conjugated antibodies against CD45, CD11b, F4/80, and CD3 for surface staining according to the manufacturer's instructions. After washing, samples were analyzed using a flow cytometer. During analysis, CD45^+^ immune cells were first gated to distinguish immune from nonimmune populations. Within the CD45^+^ compartment, monocytes and macrophages were identified as CD11b^+^ and F4/80^+^ populations, respectively, while T cells were identified as CD3^+^ cells. CD45^−^ cells were defined as nonimmune cells. OMV uptake was determined by measuring the percentage of Dio‐positive cells within each gated population.

### 4.27 Primary hMDMs

Peripheral blood mononuclear cells (PBMCs) were isolated from healthy donors by Ficoll density gradient centrifugation following Institutional Review Board approval. CD14^+^ monocytes were positively selected using magnetic beads (Miltenyi Biotec) and differentiated into macrophages in RPMI 1640 medium supplemented with 10% FBS and 50 ng/mL recombinant human M‐CSF for 7 days. M2 macrophages were generated by stimulation with recombinant human IL‐4 (20 ng/mL) and IL‐13 (20 ng/mL) for 24 h to mimic alternative activation. For TLR4 signaling inhibition, cells were pretreated with 5 µM TAK‐242 (AmBeed) for 1 h before OMV incubation.

For OMV uptake assays, polarized macrophages were incubated with Dio‐labeled siTREM2@OMVs (10 µg/mL) for 1 h at 37°C. After incubation, cells were washed twice with cold PBS and immediately processed for flow cytometry.

### 4.28 Macrophage Phagocytosis Assay

To evaluate the phagocytic capacity of macrophages following different treatments, a coculture system was established using RAW264.7 macrophages and tumor cells. RAW264.7 cells were subjected to indicated treatments and subsequently cocultured with E0771 tumor cells labeled with CFSE at a ratio of 1:2 for 4 h. After coculture, cells were washed with PBS, collected, and stained with F4/80 antibody to identify the macrophage population. The phagocytic activity was quantified by flow cytometry as the percentage of F4/80^+^CFSE^+^ double‐positive cells.

For confocal imaging experiments, RAW264.7 cells were cocultured with mCherry‐labeled E0771 cells. Following coculture, cells were fixed and stained with DAPI for nuclear visualization. Phagocytosis of tumor cells by macrophages was observed and recorded using a laser confocal microscope.

### 4.29 Bone Slice Resorption Assay

To mimic in vivo bone resorption, lyophilized bovine bone slices (Φ 6 mm × 0.5 mm) were immersed in 100 µL 0.9% NaCl solution for 2 h, followed by three washes with 0.9% NaCl solution. Bone marrow‐derived macrophages (BMMs) were seeded at a density of 2 × 10^5^ cells per well in 6‐well collagen‐coated plates (Corning Inc., Corning, NY, USA) in complete α‐MEM supplemented with M‐CSF. Then BMMs were treated with siTREM2@OMVs (1 µg/mL) for 2 h. Osteoclastogenesis was induced by 50 ng/mL RANKL for 5 days. Subsequently, cells were detached using cell dissociation solution (Sigma‐Aldrich, Australia), and 3 × 10^4^ cells were seeded onto bone slices for an additional 4 days. At the experiment's conclusion, bone slices were fixed in 4% PFA for 30 min, and surface cells were removed by brushing. The slices were then sputter‐coated with Au–Pd and examined via scanning electron microscopy (SEM; TM‐1000, Hitachi, Tokyo, Japan).

### 4.30 Transwell

Matrigel (Corning, NY, USA) was diluted 1:8 with PBS. Sixty microliters of diluted Matrigel were applied to 6.5 mm Transwell inserts with 8.0 µm pore polycarbonate membranes (Corning, NY, USA) and incubated in 24‐well plates at 37°C for 5 h. E0771 cells, prestarved in serum‐free DMEM for 12 h, were seeded in the upper chamber with 200 µL of FBS‐free culture medium. Osteoclasts, derived from BMDMs via induction with 50 ng/mL RANKL and pre‐treated with PBS or siTREM2‐loaded outer membrane vesicles (siTREM2@OMVs), were placed in the lower chamber with 750 µL of culture medium containing 10% FBS. The upper chambers were then inserted into the lower chambers and incubated for 24 h. After incubation, the medium in the upper chambers was removed, and the chambers were washed twice with PBS. Cells were fixed with 4% PFA and stained with 0.1% crystal violet. Noninvasive cells were removed using cotton swabs, and the upper chambers were observed under an inverted phase‐contrast microscope (Olympus Corporation, Tokyo, Japan).

### 4.31 In Vivo Therapeutic Efficacy

The orthotopic tumor‐bearing mouse model was established by intratibial injection of 1 × 10^6^ E0771‐luci cells suspended in 10 µL PBS into the left tibial bone marrow cavity of anesthetized mice. Mice were randomly divided into four groups (*n* = 5 per group) using a random number generator to minimize selection bias: PBS (control), OMVs, siTREM2@OMVs, siTREM2@ETP‐PEOz‐OMVs. Treatments (100 µL volume; siTREM2 dose: 5 mg/kg body weight) were administered intravenously via tail vein injection at 3 day intervals, with the first dose initiated on day 5 posttumor implantation. Tumor progression was monitored via bioluminescence imaging (AniView system) every 5 days, and tumor volume was measured every 2 days using digital calipers (formula: *V* = *a* × *b* ^2^ / 2, where a is the longest diameter and b is the shortest). Body weight was recorded daily to assess toxicity. All assessments were performed blindly by independent observers. Experiments adhered to ARRIVE guidelines and were approved by the Ethics Committee for Experimental Animals of the Second Affiliated Hospital, Zhejiang University School of Medicine. Humane endpoints included tumor volume >1000 mm^3^ or body weight loss >20%, at which point mice were euthanized via CO_2_ inhalation.

### 4.32 Histological Staining

Following nanovesicle treatment, mice were euthanized and tumors along with major organs (heart, lung, liver, spleen, kidney) were harvested for histopathological analysis. Tissues were fixed in 4% PFA for 24 h, paraffin‐embedded, and sectioned into 5 µm‐thick slices. For H&E staining, sections were dewaxed, rehydrated, and stained using standard protocols before microscopic imaging. For Ki‐67 immunohistochemistry, 5‐micrometer‐thick tumor sections were then incubated with blocking buffer for 30 min. Sections were incubated with anti‐Ki‐67 primary antibody at 4°C overnight. Next, the tumor slices were washed and incubated with secondary antibodies. DAB was used for chromogenic development, followed by hematoxylin counterstaining. Slides were imaged under a microscope.

### 4.33 RNA Sequencing

RNA sequencing and associated bioinformatics analyses were performed by Majorbio Technology Co., Ltd. (Shanghai, China). Tumor tissues from PBS‐treated control mice and siTREM2@ETP‐PEOz‐OMVs‐treated experimental mice were collected posttreatment for transcriptomic profiling. Cellular RNA was extracted using TRIzol reagent for transcriptome sequencing. RNA quality was systematically evaluated through purity assessment, concentration quantification, and integrity verification to confirm compliance with transcriptomic sequencing requirements. RNA samples meeting established quality thresholds were subsequently processed for library preparation using standard protocols, followed by high‐throughput sequencing on the Illumina NovaSeq 6000 platform with 150 bp paired‐end configuration. Bioinformatic processing was executed through the BMKCloud analytical suite, employing the Mus musculus reference genome GRCm38/mm10 for sequence alignment. Transcript abundance was normalized and expressed as Fragments Per Kilobase of transcript per Million mapped reads (FPKM) to enable cross‐sample comparisons. Differential expression analysis between groups was performed using the DESeq2 package. The False Discovery Rate (FDR) was controlled using the Benjamini–Hochberg procedure, and genes with an adjusted *p*‐value < 0.05 and |log2(fold change)| > 1.2 were defined as DEGs. GO and KEGG pathway enrichment analyses of DEGs were performed using the clusterProfiler R package, with terms having an FDR < 0.05 considered significantly enriched. GSEA was performed using GSEA software (v4.3.2) against the MSigDB hallmark and KEGG gene sets, with |NES| > 1 and *p*‐value < 0.05 considered significant.

### 4.34 Statistical Analysis

Quantitative data were expressed as mean ± standard deviation (SD), with experimental replicates explicitly indicated by *n* values in the corresponding figure legends. Normality was assessed using Shapiro–Wilk test. Intergroup comparisons between two independent cohorts were statistically analyzed using an independent‐samples *t*‐test. For experimental designs involving three or more treatment groups, one‐way analysis of variance (ANOVA) was implemented with Tukey's post‐hoc test for multiple comparisons. All statistical computations were executed in IBM SPSS Statistics software (Version 24.0) and GraphPad Prism (v9.0). *p* < 0.05 was considered statistically significant.

## Author Contributions

F.L.C. and Y.C.X. conceptualized and designed the research. F.L.C., Y.C.X., and X.M. performed the experiments. F.L.C., YC.X., X.M., and L.C. collected and processed the data. All authors analyzed and interpreted the data. F.L.C., Y.C.X., X.H.Y., and S.D.W. wrote the manuscript. All authors discussed the results and commented on the manuscript.

## Ethical Approval

This study was conducted in accordance with the ethical principles of the Declaration of Helsinki, following approval by the Ethics Committee of Human Research of the Second Affiliated Hospital, Zhejiang University School of Medicine (Approval No. (2025) Ethical Review No. (1261)). Written informed consent was obtained from all participants prior to sample acquisition.

## Conflicts of Interest

The authors declare no conflicts of interest.

## Supporting information




**Supporting File**: advs75014‐sup‐0001‐SuppMat.docx.

## Data Availability

The authors declare that all the data supporting the findings of this study are available within the article and Supplementary Information.
