# Supplementary material for: Hierarchical Targeting of TREM2+ Myeloid Cells via Acid‐Triggered OMVs Reprogram Immunosuppression and Suppress Osteolysis in Bone‐Metastatic TNBC
Source: Adv Sci (Weinh). 2026 Apr 7;13(32):e17369. doi: 10.1002/advs.202517369 (PMC13252654; doi:10.1002/advs.202517369)
Supplement: Supplementary file 1 — Supporting File: advs75014‐sup‐0001‐SuppMat.docx. [file ADVS-13-e17369-s001.docx]

### **Supporting Information**

Hierarchical targeting of TREM2^+^ Myeloid Cells via Acid-Triggered OMVs Reprogram Immunosuppression and Suppress Osteolysis in Bone-Metastatic TNBC

Fanglu Chen^1,2,3†^, Yucheng Xue^1,2,3†^, Xiao Ma^1,2,3†^, Liang Chen^1,2,3^, Zhenxuan Shao^1,2,3^, Hangxiang Sun^1,2,3^, Haochen Mou^1,2,3^, Fangqian Wang^1,2,3^, Zilong Li^1,2,3^, Yechao Shen^1,2,3^, Zhaoming Ye^1,2,3*^, Xiaohua Yu^1,2,3*^, Shengdong Wang^1,2,3*^

^†^These authors contributed equally to this work.

^1^ Musculoskeletal Tumor Center, Department of Orthopedics, The Second Affiliated Hospital of Zhejiang University School of Medicine, Hangzhou 310009, P.R. China.

^2^ Institute of Orthopedic Research, Zhejiang University, Hangzhou 310009, P.R. China

^3^ Key Laboratory of Motor System Disease Research and Precision Therapy of Zhejiang Province, Hangzhou, Zhejiang, China.

^*^**Corresponding author**:

Zhaoming Ye^*^, E-mail: yezhaoming@zju.edu.cn

Xiaohua Yu^*^, E-Mail: xiaohuayu@zju.edu.cn

Shengdong Wang^*^, E-mail: wangshengdong@zju.edu.cn

**Contents:**

**Figure S1** Dot plot of cell type-identifying genes in identified myeloid cluster types.

**Figure S2** Immunohistochemical analysis of Ki67 expression in tumors. Scale bars: 10 μm.

**Figure S3** CD4 and CD8 fluorescent staining of tumors extracted from the mice, scale bars: 25 μm.

**Figure S4** Flow cytometric analysis and graphs of immune cell populations within the TME in mice bearing orthotopic TNBC bone metastases (n = 4 biologically independent experiments). (A) Representative contour plots and bar graph quantification for iNOS^+^ macrophages. (B) Representative contour plots and bar graph quantification for Arg1^+^ macrophages. (C) Representative contour plots with percentages for CD8^+^ T cells and CD4^+^ T cells. (D) Representative contour plots with percentages for Ly6G^-^Ly6C^+^ cells (M-MDSCs). Data were presented as the mean ± SD (unpaired two-tailed Student’s t-test. ***P < 0.001, ****P < 0.0001).

**Figure S5** iNOS and Arg1 fluorescent staining of tumors extracted from the mice. Scale bars: 50 μm.

**Figure S6** The gating strategy for flow cytometry analysis. (**A**) Gating strategy for iNOS^+^ and Arg1^+^ macrophages in intratibial TNBC bone metastasis mice. (**B**) Gating strategy for T cells in in intratibial TNBC bone metastasis mice. (**C**) Gating strategy for M-MDSCs in intratibial TNBC bone metastasis mice. (**D**) Gating strategy for TLR4 expression, OMV uptake, CD86, and CD206 in primary human monocyte-derived macrophages across differentiation stages. (**E**) Gating strategy for identifying immune and non-immune cell populations in tumor tissues. (**F**) Gating strategy for macrophage polarization. (**G**) Gating strategy for phagocytosis assay. (**H**) Gating strategy for Trem2^+^ myeloid cells.

**Figure S7** Flow cytometric analysis of iNOS^+^ macrophages, Arg1^+^ macrophages, CD8^+^ T cells, CD4^+^ T cells, and Ly6c^+^Ly6G^-^ M-MDSCs within the TME (n = 4 biologically independent experiments). Data were presented as the mean ± SD (unpaired two-tailed Student’s t-test, ns, no significance.).

**Figure S8** The mass spectrum showed the successful synthesis of Peptide A, Peptide B and Peptide C.

**Figure S9** Fluorescence staining to determine the binding affinity of ETP in vitro and fluorescence intensity analysis of Cy5.5-peptide dyes stained with E0771 cells (n = 3 biologically independent experiments). Scale bars: 50 μm.

**Figure S10** Representative confocal images showing the specificity of OMVs in RAW264.7 cells. RAW264.7 cells, E0771 cells, and 3T3L1 cells were costained by DAPI and FITC-phalloidin after 1 h-incubation with Did-labeled OMVs. Scale bars: 25 μm.

**Figure S11** Synthetics route of DSPE-PEOz-ETP.

**Figure S12** ^1^H NMR of DSPE-PEOz-ETP.

**Figure S13** The ETP anchor capacity of DSPE-PEOz-ETP on OMVs (n = 3 biologically independent experiments).

**Figure S14** FTIR spectra of DSPE-PEOz-ETP before (pH 7.4, black line) and after (pH 5.4 for 30 min, red line) acid exposure, demonstrating pH-responsive cleavage. Key spectral changes include a marked reduction in the amide C=O stretch peak at ~1640 cm⁻¹ and the emergence of a broad O-H stretch at ~3400 cm⁻¹, indicative of acid-triggered hydrolysis products.

**Figure S15** Viabilities of after the incubation with different concentrations of OMVs for 24 h (n = 5 biologically independent samples).

**Figure S16** Quantification of siTREM2@OMVs and siTREM2@ETP-PEOz-OMVs fluorescence signal of whole blood collected from mice bearing orthotopic TNBC bone metastases at fixed time points.

**Figure S17** Fluorescence images and quantitative analysis of the fluorescence intensity in the major organs and tumor sites after siTREM2@OMVs or siTREM2@ETP-PEOz-OMVs 24h and 72h after intravenous injection. siTREM2@OMVs and siTREM2@ETP-PEOz-OMVs were labeled with Did (n = 3 biologically independent experiments). Data were presented as the mean ± SD (unpaired two-tailed Student’s t-test. *P < 0.05, ****P < 0.0001).

**Figure S18** TLR4 expression and OMV uptake across differentiation and polarization stages of primary human monocyte-derived macrophages (n = 3 biologically independent donors). Representative flow cytometry contour plots and quantification showing TLR4 expression (A, B) and OMV uptake (C, D) in freshly isolated monocytes, mature macrophages and M2 macrophages. Data are mean ± SD (one-way ANOVA comparisons tests, ns, not significant, ****P < 0.0001). Gating strategies as in Figure S6D.

**Figure S19** TLR4 signaling inhibition by TAK-242 blocks OMV uptake in primary human monocytes and macrophages (n = 3 biologically independent donors). Representative contour plots (left) and quantification (right) of OMV-FITC⁺/CD11b⁺ cells after 1 h pretreatment with 5 μM TAK-242 or vehicle control. Data are mean ± SD (unpaired two-tailed Student’s t-test, **P < 0.01, ***P < 0.001). Gating strategies as in Figure S6D.

**Figure S20** In vivo cellular distribution of Dio-labeled siTREM2@ETP-PEOz-OMVs in tibial tumor tissues. Representative flow cytometry plots and quantification of the percentage of OMV⁺ cells showing uptake of Dio-labeled OMVs in different cell populations isolated from tibial tumor tissues of E0771 bone metastasis–bearing mice 24 h after intravenous administration of Dio-labeled siTREM2@ETP-PEOz-OMVs, including CD11b⁺ monocytes, F4/80⁺ macrophages, CD3⁺ T cells, and CD45⁻ non-immune cells. Data are mean ± SD (one-way ANOVA comparisons tests, ****P < 0.0001). Gating strategies as in Figure S6E.

**Figure S21** Confocal imaging of RAW 264.7 cells co-stained with Cy3-labeled siTREM2 (red) and a lysosomal tracer dye (green) demonstrates that OMVs loaded with Cy3-siTREM2 can achieve lysosomal escape.

**Figure S22** siTREM2@OMVs repolarize primary human macrophages toward a pro-inflammatory phenotype (n = 3 biologically independent donors). Representative flow cytometry contour plots (A, C) and quantification (B, D) of CD86⁺ and CD206⁺ populations in CD11b⁺/CD68⁺ macrophages after 24h treatment with siTREM2@OMVs versus control. Data are mean ± SD (unpaired two-tailed Student’s t-test, **P < 0.01, ***P < 0.001). Gating strategies as in Figure S6D.

**Figure S23** Differentially expressed gene (DEG) heatmap analysis of the control group and siTREM2@OMVs group. Blue indicates downregulated genes. Red indicates upregulated genes.

**Figure S24** Hematological analysis after treatment. The level of red blood cells (RBC), white blood cells (WBC), platelets (PLT), alanine aminotransferase (ALT), aspartate aminotransferase (AST), creatine kinase (CK), creatinine (CR), and total protein (TP) between the treated group and the control group are detected after 10 days of treatment (n = 4 biologically independent experiments). Data were presented as the mean ± SD (unpaired two-tailed Student’s t-test. ns, no significance).

**Figure S25** H&E staining of major organs at the endpoint of the antitumor experiment against TNBC. The experiments were repeated three times independently.

**Figure S26** DEG heatmap analysis of the control group and siTREM2@ETP-PEOz-OMVs treated group. Blue indicates downregulated genes. Red indicates upregulated genes.

**Figure S27** RT-qPCR analysis of Tnf, S100a8, Spp1, Stat1, Nfkbia, Irf8, Myd88 and Cxcl13 expression in cancer cells sorted from bone metastases of PBS or siTREM2@ETP-PEOz-OMVs treated group (n = 3 biologically independent experiments). Data were presented as the mean ± SD (unpaired two-tailed Student’s t-test, *P < 0.05, **P < 0.01, ***P < 0.001, ****P < 0.0001).

**Figure S28** Control staining with AF488- or AF594-conjugated secondary antibodies alone (without primary antibodies) confirmed the absence of non-specific binding.

**Table 1** Summary of peptide sequences and frequencies in the first and third round of phage display against E0771 tumors.

**Figure S1** Dot plot of cell type-identifying genes in identified myeloid cluster types.

Ki67

Trem2^-/-^

WT

**Figure S2** Immunohistochemical analysis of Ki67 expression in tumors. Scale bars: 10 μm.

Trem2^-/-^

WT

CD4

CD8

**Figure S3** CD4 and CD8 fluorescent staining of tumors extracted from the mice. Scale bars: 25 μm.

**Figure S4** Flow cytometric analysis and graphs of immune cell populations within the TME in mice bearing orthotopic TNBC bone metastases (n = 4 biologically independent experiments). (**A**) Representative contour plots and bar graph quantification for iNOS^+^ macrophages. (**B**) Representative contour plots and bar graph quantification for Arg1^+^ macrophages. (**C**) Representative contour plots with percentages for CD8^+^ T cells and CD4^+^ T cells. (**D**) Representative contour plots with percentages for Ly6G^-^Ly6C^+^ cells (M-MDSCs). Data were presented as the mean ± SD (unpaired two-tailed Student’s t-test. ***P < 0.001, ****P < 0.0001).

**Figure S5** iNOS and Arg1 fluorescent staining of tumors extracted from the mice. Scale bars: 50 μm.

**Figure S6** The gating strategy for flow cytometry analysis. (**A**) Gating strategy for iNOS^+^ and Arg1^+^ macrophages in intratibial TNBC bone metastasis mice. (**B**) Gating strategy for T cells in in intratibial TNBC bone metastasis mice. (**C**) Gating strategy for M-MDSCs in intratibial TNBC bone metastasis mice. (**D**) Gating strategy for TLR4 expression, OMV uptake, CD86, and CD206 in primary human monocyte-derived macrophages across differentiation stages. (**E**) Gating strategy for identifying immune and non-immune cell populations in tumor tissues. (**F**) Gating strategy for macrophage polarization. (**G**) Gating strategy for phagocytosis assay. (**H**) Gating strategy for Trem2^+^ myeloid cells.

**Figure S7** Flow cytometric analysis of iNOS^+^ macrophages, Arg1^+^ macrophages, CD8^+^ T cells, CD4^+^ T cells, and Ly6c^+^Ly6G^-^ M-MDSCs within the TME (n = 4 biologically independent experiments). Data were presented as the mean ± SD (unpaired two-tailed Student’s t-test, ns, no significance.).

**Figure S8** The mass spectrum showed the successful synthesis of Peptide A, Peptide B and Peptide C.

Peptide C

Peptide B

Peptide A

**Figure S9** Fluorescence staining to determine the binding affinity of ETP in vitro and fluorescence intensity analysis of Cy5.5-peptide dyes stained with E0771 cells. Peptide A, Peptide B, and Peptide C were conjugated to maleimide-tagged Cy5.5 to form Cy5.5-peptide dyes, which were added to the medium of E0771 cells (n = 3 biologically independent experiments). Scale bars: 50 μm. Data were presented as the mean ± SD (one-way ANOVA comparisons tests, **P < 0.01).

Peptide A

Peptide B

Peptide C

Cy5.5 dyes

FITC-Phalloidin

DAPI

MERGE

DID

DAPI

MERGE

FITC-Phalloidin

3T3L1

E0771

RAW264.7

**Figure S10** Representative confocal images showing the specificity of OMVs in RAW264.7 cells. RAW264.7 cells, E0771 cells, and 3T3L1 cells were costained by DAPI and FITC-phalloidin after 1 h-incubation with Did-labeled OMVs. Scale bars: 25 μm.

**Figure S11** Synthetics route of DSPE-PEOz-ETP.

**Figure S12** ^1^H NMR of DSPE-PEOz-ETP.

**Figure S13** The ETP anchor capacity of DSPE-PEOz-ETP on OMVs (n = 3 biologically independent experiments). Data were presented as the mean ± SD.

**Figure S14** FTIR spectra of DSPE-PEOz-ETP before (pH 7.4, black line) and after (pH 5.4 for 30 min, red line) acid exposure, demonstrating pH-responsive cleavage. Key spectral changes include a marked reduction in the amide C=O stretch peak at ~1640 cm⁻¹ and the emergence of a broad O-H stretch at ~3400 cm⁻¹, indicative of acid-triggered hydrolysis products.

**Figure S15** Viabilities of after the incubation with different concentrations of OMVs or siTREM2@ETP-PEOz-OMVs for 24 h (n = 5 biologically independent experiments). Data were presented as the mean ± SD.

**Figure S16** Pharmacokinetics analysis of fluorescently labeled siTREM2@OMVs and siTREM2@ETP-PEOz-OMVs in whole blood from mice bearing orthotopic TNBC bone metastases. (A) Representative fluorescence images of blood samples collected at fixed time points (0.5 h, 3 h, 6h, 12 h, 24 h) post-intravenous administration. (B) Quantitative plot of mean fluorescence signal intensity over time, with error bars representing standard deviation (n = 3 biologically independent experiments).

**Figure S17** Fluorescence images (**A**) and quantitative analysis (**B**) of the fluorescence intensity in the major organs and tumor sites after siTREM2@OMVs or siTREM2@ETP-PEOz-OMVs 24h and 72h after intravenous injection. siTREM2@OMVs and siTREM2@ETP-PEOz-OMVs were labeled with Did (n = 3 biologically independent experiments). Data were presented as the mean ± SD (unpaired two-tailed Student’s t-test. *P < 0.05, ****P < 0.0001).

**Figure S18** TLR4 expression and OMV uptake across differentiation and polarization stages of primary human monocyte-derived macrophages (n = 3 biologically independent donors). Representative flow cytometry contour plots and quantification showing TLR4 expression (A, B) and OMV uptake (C, D) in freshly isolated monocytes, mature macrophages and M2 macrophages. Data are mean ± SD (one-way ANOVA comparisons tests, ns, not significant, ****P < 0.0001). Gating strategies as in Figure S6D.

**Figure S19** TLR4 signaling inhibition by TAK-242 blocks OMV uptake in primary human monocytes and macrophages (n = 3 biologically independent donors). Representative contour plots (left) and quantification (right) of OMV-FITC⁺/CD11b⁺ cells after 1 h pretreatment with 5 μM TAK-242 or vehicle control. Data are mean ± SD (unpaired two-tailed Student’s t-test, **P < 0.01, ***P < 0.001). Gating strategies as in Figure S6D.

**Figure S20** In vivo cellular distribution of Dio-labeled siTREM2@ETP-PEOz-OMVs in tibial tumor tissues. Representative flow cytometry plots and quantification of the percentage of OMV⁺ cells showing uptake of Dio-labeled OMVs in different cell populations isolated from tibial tumor tissues of E0771 bone metastasis–bearing mice 24 h after intravenous administration of Dio-labeled siTREM2@ETP-PEOz-OMVs, including CD11b⁺ monocytes, F4/80⁺ macrophages, CD3⁺ T cells, and CD45⁻ non-immune cells. Data are mean ± SD (one-way ANOVA comparisons tests, ****P < 0.0001). Gating strategies as in Figure S6E.

**Figure S21** Confocal laser scanning microscopy of RAW 264.7 cells demonstrating lysosomal escape of Cy3-labeled siTREM2 (red) delivered via OMVs, with co-staining using LysoTracker (green) for lysosomes and DAPI (blue) for nuclei. Dashed white lines indicate the path for fluorescence intensity profiling. (**A**) Cells treated with siTREM2@OMVs for 3 h, showing substantial co-localization between siTREM2 and lysosomal signals in the intensity profile. (**B**) Cells treated with siTREM2@OMVs for 6 h, exhibiting reduced co-localization and evidence of lysosomal escape, as peaks in the intensity profile are separated.

**Figure S22** siTREM2@OMVs repolarize primary human macrophages toward a pro-inflammatory phenotype (n = 3 biologically independent donors). Representative flow cytometry contour plots (A, C) and quantification (B, D) of CD86⁺ and CD206⁺ populations in CD11b⁺/CD68⁺ macrophages after 24h treatment with siTREM2@OMVs versus control. Data are mean ± SD (unpaired two-tailed Student’s t-test, **P < 0.01, ***P < 0.001). Gating strategies as in Figure S6D.

**Figure S23** Differentially expressed gene (DEG) heatmap analysis of the control group and siTREM2@OMVs group. Blue indicates downregulated genes. Red indicates upregulated genes.

**Figure S24** Hematological analysis after treatment. The level of red blood cells (RBC), white blood cells (WBC), platelets (PLT), alanine aminotransferase (ALT), aspartate aminotransferase (AST), creatine kinase (CK), creatinine (CR), and total protein (TP) between the treated group and the control group are detected after 10 days of treatment (n = 4 biologically independent experiments). Data were presented as the mean ± SD (unpaired two-tailed Student’s t-test. ns, no significance).

**Figure S25** H&E staining of major organs at the endpoint of the antitumor experiment against TNBC. The experiments were repeated three times independently.

Control

SiTREM2@ETP-PEOz-OMVs

heart

Liver

Lung

Spleen

Kidney

**Figure S26** DEG heatmap analysis of the control group and siTREM2@ETP-PEOz-OMVs treated group. Blue indicates downregulated genes. Red indicates upregulated genes.

**Figure S27** RT-qPCR analysis of Tnf, S100a8, Spp1, Stat1, Nfkbia, Irf8, Tlr8 and Cxcl13 expression in cancer cells sorted from bone metastases of PBS or siTREM2@ETP-PEOz-OMVs treated group (n = 3 biologically independent experiments). Data were presented as the mean ± SD (unpaired two-tailed Student’s t-test, *P < 0.05, **P < 0.01, ***P < 0.001, ****P < 0.0001).

**Figure S28** Control staining with AF488- or AF594-conjugated secondary antibodies alone (without primary antibodies) confirmed the absence of non-specific binding. Scale bars: 50 μm.

| Frequency | | | | |
| --- | --- | --- | --- | --- |
| Peptide sequence | Name | First round  [19 clones] | Third round  [13 clones] | Sum  [32 clones] |
| TIPNLTRVSNIV | Peptide A | 7/19 | 7/13 | 14/32 |
| GTNWSIHENNMG | Peptide B | 4/19 | 2/13 | 6/32 |
| SWSNASDYHIGA | Peptide C | 1/19 | 2/13 | 3/32 |
| TLHPGYSLKMGD | Peptide D | 1/19 | 1/13 | 2/32 |
| YFCASCSQIVLH | Peptide E | 1/19 | 1/13 | 2/32 |
| NPRSDTASVGVW | Peptide F | 1/19 | 1/13 | 2/32 |
| RLVSEPSLYPPS | Peptide G | 1/19 | 0 | 1/32 |
| AFERSMPFLGLT | Peptide H | 1/19 | 0 | 1/32 |
| FPWPATDVIGNI | Peptide I | 1/19 | 0 | 1/32 |
| NFRFEESYVMRF | Peptide J | 1/19 | 0 | 1/32 |
| CYSSAKDEISRV | Peptide K | 1/19 | 0 | 1/32 |

**Table 1** Summary of peptide sequences and frequencies in the first and third round of phage display against E0771 tumors.
